# Epithelial cell plasticity drives endoderm formation during gastrulation

**DOI:** 10.1038/s41556-021-00694-x

**Published:** 2021-06-24

**Authors:** Katharina Scheibner, Silvia Schirge, Ingo Burtscher, Maren Büttner, Michael Sterr, Dapeng Yang, Anika Böttcher, Ansarullah, Martin Irmler, Johannes Beckers, Filippo M. Cernilogar, Gunnar Schotta, Fabian J. Theis, Heiko Lickert

**Affiliations:** 1 Institute of Diabetes and Regeneration Research, Helmholtz Diabetes Center, Helmholtz Zentrum München, Munich, Germany; 2 Institute of Stem Cell Research, Helmholtz Zentrum München, Munich, Germany; 3 German Center for Diabetes Research (DZD), Munich, Germany; 4 Institute of Computational Biology, Helmholtz Zentrum München, Munich, Germany; 5 Developmental Biology Program, Sloan Kettering Institute, New York, NY USA; 6 Institute of Experimental Genetics, Helmholtz Zentrum München, Munich, Germany; 7 School of Life Sciences Weihenstephan, Technische Universität München, Freising, Germany; 8 Division of Molecular Biology, Biomedical Center, Faculty of Medicine, Ludwig Maximilian University, Munich, Germany; 9 Department of Mathematics, Technische Universität München, Munich, Germany; 10 School of Life Sciences Weihenstephan, Technische Universität München, Freising, Germany; 11 School of Medicine, Klinikum Rechts der Isar, Technische Universität München, Munich, Germany

**Keywords:** Embryology, Embryonic stem cells, Gastrulation, Epithelial-mesenchymal transition, Cell lineage

## Abstract

It is generally accepted that epiblast cells ingress into the primitive streak by epithelial-to-mesenchymal transition (EMT) to give rise to the mesoderm; however, it is less clear how the endoderm acquires an epithelial fate. Here, we used embryonic stem cell and mouse embryo knock‐in reporter systems to combine time-resolved lineage labelling with high-resolution single-cell transcriptomics. This allowed us to resolve the morphogenetic programs that segregate the mesoderm from the endoderm germ layer. Strikingly, while the mesoderm is formed by classical EMT, the endoderm is formed independent of the key EMT transcription factor Snail1 by mechanisms of epithelial cell plasticity. Importantly, forkhead box transcription factor A2 (Foxa2) acts as an epithelial gatekeeper and EMT suppressor to shield the endoderm from undergoing a mesenchymal transition. Altogether, these results not only establish the morphogenetic details of germ layer formation, but also have broader implications for stem cell differentiation and cancer metastasis.

## Abstract (web summary)

Scheibner et al. demonstrate that, during gastrulation in the mouse, epithelial epiblast progenitors upregulate Foxa2 and form the definitive endoderm independently of a full EMT–MET cycle.

## Main

The recent single-cell genomics revolution has generated transcriptional and epigenetic roadmaps for the formation of the three principal germ layers during gastrulation^1–3^. Epithelial-to-mesenchymal transition (EMT) and mesenchymal-to-epithelial transition (MET) are evolutionarily conserved processes that occur during development and are essential for gastrulation and embryonic morphogenesis, but if they are dysregulated in adulthood they lead to cancer metastasis^4–6^. Although gastrulation serves as the mechanistic basis to understand EMT, cancer metastasis, stem cell differentiation and congenital disease, its understanding is incomplete in mammals. During gastrulation, epiblast cells exit pluripotency and allocate to one of the three germ layers—the ectoderm, mesoderm or definitive endoderm lineage—to generate the progenitors of major organs in the body^7^. The T-box transcription factor Brachyury (T) and forkhead box transcription factor A2 (Foxa2) are master regulators of mesoderm and endoderm formation, respectively^8–11^. Morphogen gradients along the anterior–posterior axis induce posterior epiblast cells to undergo EMT and ingress into the primitive streak region (the space between the epiblast and visceral endoderm epithelial layers)^7,12,13^. Upon high Wnt/β-catenin, transforming growth factor-β (TGF-β) and fibroblast growth factor signalling, EMT transcription factors such as Mesp1 and 2 (refs. ^14,15^), Twist1 (ref. ^16^), Snail1 and 2 (refs. ^16–18^) and Zeb1 and 2 (refs. ^19,20^) are induced. Following the activation of an EMT program, columnar-shaped epiblast cells lose their epithelial morphology by dynamic expression changes and remodelling of apical–basal polarity and cell–cell adhesion. Simultaneously, cells adopt a mesenchymal fate by downregulation of E-cadherin and by upregulation of N-cadherin, vimentin and α-smooth muscle actin, resulting in ingression and migration of mesodermal cells into the primitive streak—classical hallmarks of EMT^17,18,21–23^. It is generally assumed that definitive endoderm (DE) progenitors undergo a further MET to give rise to the epithelial endoderm layer, in an EMT–MET cycle^12,13,24–28^; however, this idea is based on *Drosophila* and zebrafish model systems and has never been formally proven in mammals^13^. Previously, we noticed that before primitive streak formation and initiation of gastrulation, proximal T^+^ mesoderm and distal Foxa2^+^ endoderm progenitors are already segregated in the epiblast^11^. As Foxa2 regulates axial mesendoderm (AME) and definitive endoderm differentiation, cell polarity and epithelialization^11^, we hypothesized that DE progenitors leave the epiblast to form the mature definitive endoderm lineage by a process independent of a complete EMT–MET cycle.

## Results

### Definitive endoderm is formed in the absence of an EMT–MET cycle

**Fig. 1:**
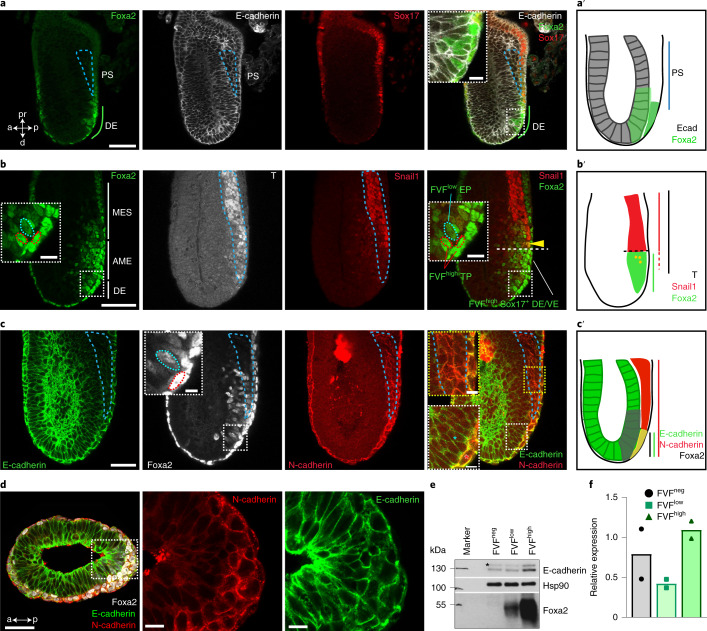
Endoderm progenitors do not show hallmarks of an EMT. **a**, Mid-streak-stage FVF/SCF embryos stained for Venus (Foxa2), E-cadherin and RFP (Sox17). The blue dashed line indicates the primitive streak (PS). **a**, anterior; d, distal; DE, definitive endoderm; p, posterior; pr, proximal. **b**, Immunohistochemistry of a mid-streak-stage FVF embryo stained for Venus (Foxa2), Snail1 and T. The blue dashed lines in the insets mark FVF^low^ epiblast progenitors (EPs) and the red dashed lines mark FVF^high^ transitory progenitors (TPs). The yellow arrowhead marks AME cells that synthesize Foxa2, T and Snail1. The horizontal white dashed line indicates the border of Snail1 expression. MES, mesoderm; TP, transitory progenitor; VE, visceral endoderm. **c**, Mid-streak-stage FVF embryo immunostained for Venus (Foxa2; white), N-cadherin and E-cadherin. The blue asterisk (rightmost image inset in white dashed box) and inset blue dashed line (inset in image second from left) mark FVF^low^ epiblast progenitors in the epiblasts, whereas the red asterisk (rightmost image inset in white dashed box) and red dashed line (inset in image second from left) indicate FVF^high^ transitory progenitors that express E-cadherin and N-cadherin. The primitive streak region is indicated by N-cadherin expression (blue dashed line in rightmost image inset in dashed yellow box). **a′**–**c′**, Depiction of Foxa2 (**a′**), T and Snail1 (**b′**) and E-cadherin and N-cadherin expression (**c′**) in gastrulating embryos based on the embryos in **a**–**c**. **d**, Transverse section through the epiblast of a mid-streak-stage wild-type embryo immunostained for Foxa2, E-cadherin and N-cadherin. **e**,**f**, Western blot analysis (**e**) and quantification (**f**) of E-cadherin from FACS-sorted FVF^neg^, FVF^low^ and FVF^high^ cells of 122 and 36 FVF embryos (*n* = 2). The asterisk marks unspecific bands. All shown confocal images are single *z* planes of a z stack. The images in **a**–**d** are representative of eight, three, three and three embryos, respectively. All samples were derived from biologically independent experiments. The data are presented as mean values. Scale bars, 50 µm (insets, 10 µm). Source data

**Fig. 2:**
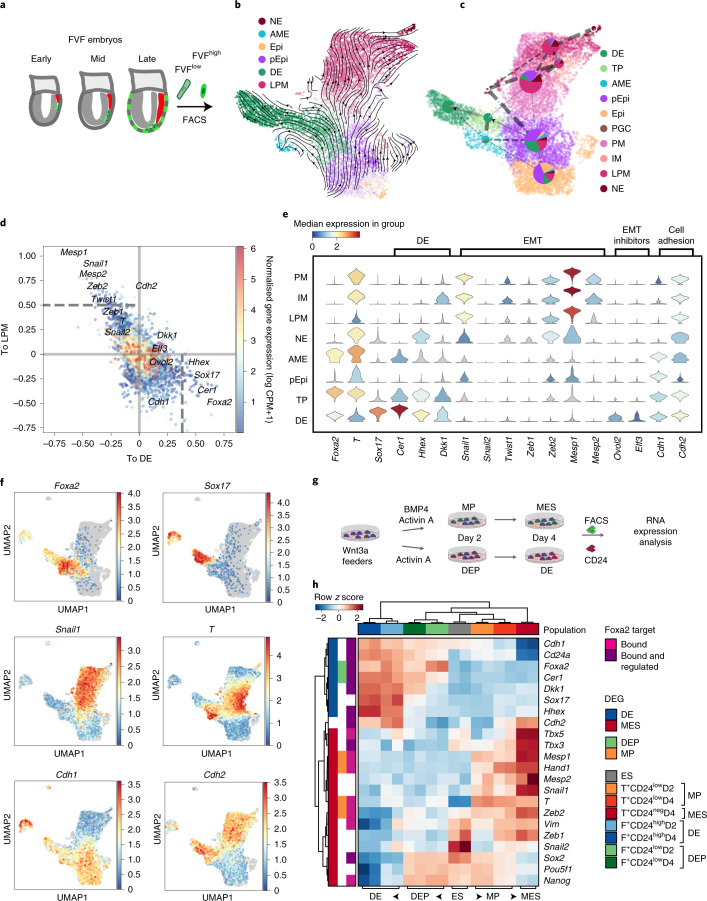
Mesoderm and endoderm form by distinct molecular programs. **a**, Schematic of FACS of early-, mid- and late-streak-stage FVF embryos for scRNA-seq analysis (*n* = 79 for early- to mid-streak-stage embryos and *n* = 24 for mid- to late-streak-stage embryos). **b**, UMAP plot with RNA velocity arrows, coloured by CellRank’s metastable state assignment. Each shown tissue is either in the initial (epiblast (Epi)), intermediate (posterior epiblast (pEpi)) or final state (AME, definitive endoderm, lateral plate mesoderm (LPM) and nascent endothelium (NE)). **c**, UMAP showing CellRank’s fate probabilities for different tissues as pie charts (*n* = 9,794 cells). The partitions of each pie chart show the previously identified initial, intermediate or final state. Dashed lines indicate significant connections between clusters (PAGA graph model). Arrows indicate consistent RNA velocity between two clusters. The thickness of each line shows the confidence of the model. The solid line without an arrowhead suggests a transition along the velocity between clusters but not unique flow. IM, intermediate mesoderm; PGC, primordial germ cell; PM, paraxial mesoderm. **d**, Scatter plot of lineage drivers, showing the correlation of gene expression for the lineages definitive endoderm and LPM, computed using CellRank. The top 50 correlated genes are indicated by dashed horizontal and vertical lines. **e**, Stacked violin plots showing the gene expression distribution (columns) with definitive endoderm, EMT, EMT inhibitors and cell adhesion genes of all tissues (rows) (*n* = 2,215 (posterior epiblast); *n* = 2,198 (paraxial mesoderm); *n* = 1,183 (LPM); *n* = 701 (definitive endoderm); *n* = 389 (intermediate mesoderm); *n* = 350 (transitory progenitors); *n* = 278 (AME); *n* = 91 (nascent endothelium)). The colours correspond to normalized median gene expression for each group. **f**, UMAP plots coloured by the log[counts per million + 1] normalized gene expression. **g**, Schematic of endoderm and mesoderm differentiation of T^GFP/+^; Foxa2^tagRFP/+^ mESCs. **h**, Heatmap of FACS-sorted endodermal and mesodermal subpopulations expressing different levels of CD24 at days 2 and 4, showing RNA expression levels of pluripotency, endoderm, mesoderm and EMT genes in mESCs (ES), early (definitive endoderm progenitor (DEP)/mesoderm progenitor (MP)) and late (definitive endoderm (DE)/mesoderm (MES)) endoderm and mesoderm cells. The coloured boxes indicate differentially expressed genes (DEGs) in DEP versus mesoderm progenitor (green and orange, respectively) or definitive endoderm versus mesoderm (blue and red, respectively) and whether Foxa2 binds (pink) or binds and regulates them (purple). Source data

**Extended Data Fig. 1:**
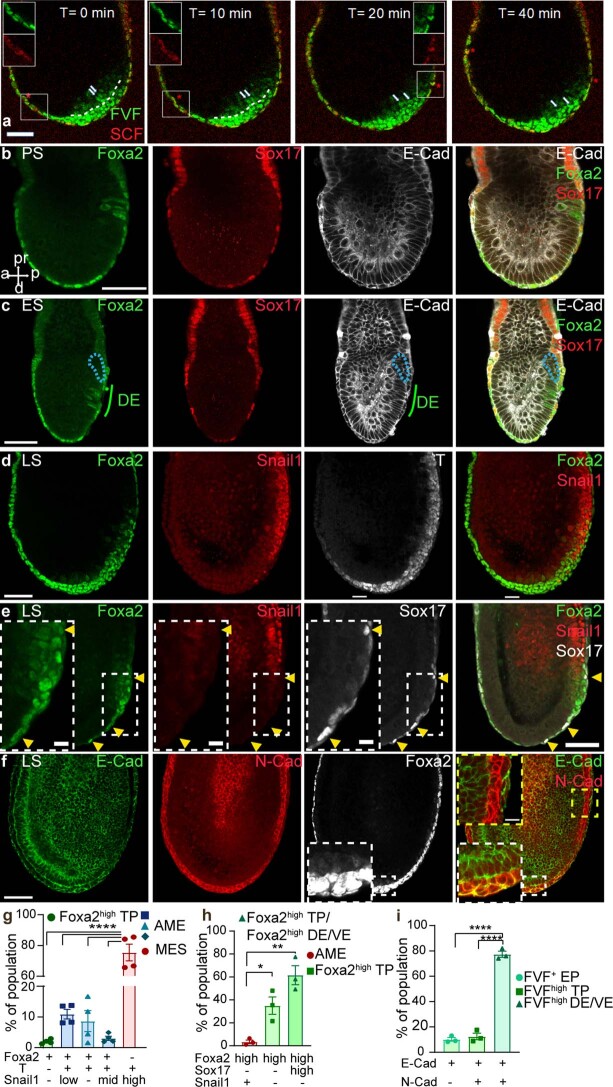
The endoderm is formed independent of a full EMT-MET cycle. **a**, Still images extracted from live cell imaging of a LS stage FVF/SCF embryo (representative of 4 embryos). The images show a single z-plane of the merge of the FVF channel in green and SCF channel in red. White dashed line shows the border of the epiblast, separating FVF^low^ and FVF^high^ expressing cells. The yellow arrows show a single FVF cell that increases fluorescent intensity; red asterisks: DE progenitor upregulating Sox17 and intercalating into the DE layer. **b**, Immunohistochemistry of E6.25 (pre-streak) (2 embryos) and E6.5 (early-streak stage) (8 embryos) FVF/SCF embryos stained for Venus (Foxa2), E-cadherin and RFP (Sox17) showing Foxa2 expressing cells appearing before PS is present and (**c**) after PS induction (8 embryos) Foxa2 expressing cells are distal to the PS (blue dashed line indicates PS). **d**,**e**, Immunohistochemistry of LS stage FVF embryo (7 embryos) stained for Venus (Foxa2), Snail1 and T (**d**), LS stage wildtype embryo (3 embryos) stained for Foxa2, Snail1 and Sox17. Note, Foxa2^high^/Sox17^+^ TP does not express Snail1 (yellow arrowhead) (**e**). **f**, LS stage FVF embryo (3 embryos) immunostained for Venus (Foxa2), N-cadherin and E-cadherin. **g**, Quantification of Foxa2, T and Snail1 positive cells in MES, AME, Foxa2^high^ TP of MS stage embryos (Ordinary one-way ANOVA with Tukey’s multiple comparison test, n = 4 embryos). **h**, Quantification of Foxa2, Sox17 and Snail1 expression in AME, Foxa2^high^ TP/Foxa2^high^ DE/VE and Foxa2^high^ TP in MS stage embryos (Ordinary one-way ANOVA with Tukey’s multiple comparison test, n = 3 embryos). **i**, Quantification of E-cadherin and N-cadherin expression in FVF^+^, FVF^high^ TP, FVF^high^ DE/VE of MS stage embryos (Ordinary one-way ANOVA with Tukey’s multiple comparison test, n = 3 embryos). All samples are derived from biologically independent experiments. Data are presented as mean values ±SEM.; ****P < 0.0001, **P < 0.0019, *P < 0.0337. Statistically non-significant results are not indicated in the figure. All shown confocal images are single planes of a z-stack unless otherwise stated. Scale bar: 50 µm, insets 10 µm. Source data

To test this hypothesis and specifically study endoderm differentiation and morphogenesis in more detail, we performed time-resolved and simultaneous lineage labelling and tracking using Foxa2–Venus fusion (FVF) and Sox17–mCherry fusion (SCF) double knock‐in reporter mouse embryonic stem cells (mESCs) and mouse embryos^29,30^. During gastrulation, FVF^low^ epiblast progenitors upregulate FVF reporter activity while they leave the epithelium and give rise to FVF^high^ transitory progenitors (Extended Data Fig. 1a and Supplementary Video 1). FVF^high^ transitory progenitors migrate between the epiblast and visceral endoderm layers until they upregulate SCF and intercalate into the outside visceral endoderm and give rise to the FVF^high^/SCF^+^ definitive endoderm lineage^11,29,30^. We recently noticed that it only takes ~12 h for FVF/SCF mESCs to differentiate from FVF^low^ progenitors into FVF^high^/SCF^+^ definitive endoderm^30^. This short timespan seems insufficient for a cell to down- and upregulate molecular machineries on the messenger RNA (mRNA) and protein level to undergo a complete EMT–MET cycle. Thus, we investigated first whether Foxa2^low^ epiblast progenitors leave the epithelium by EMT in early-, mid- and late-streak-stage embryos. Before gastrulation, FVF^low^ epiblast progenitors are found in the posterior epiblast region, suggesting that epiblast cells are already fate specified (Extended Data Fig. 1b)^11^. During primitive streak induction and elongation, FVF^low^ epiblast progenitors occupy an epiblast domain distal to the morphological anterior primitive streak (APS) region (Fig. 1a,a′ and Extended Data Fig. 1c). Within the FVF^low^ epiblast domain, single FVF^high^ transitory progenitors appear, leave the epithelium and squeeze as elongated cells between the epiblast and visceral endoderm (Fig. 1a–c, insets)^11,17^. This is in stark contrast with the proximal primitive streak, where T^+^ mesoderm cells with a mesenchymal morphology span several cell diameters across the primitive streak region (Fig. 1a–c). During gastrulation, mesoderm formation is driven by the key EMT transcription factor Snail1 (refs. ^17,18^). At the mid- to late-streak stage, a distinct separation of three populations in the primitive streak region was apparent (see the schematic in Fig. 1b′): a large T^+^ mesoderm population in the proximal primitive streak region; a few Foxa2^high^/T^+^ AME progenitors (Fig. 1b,b′); and Foxa2^low^ epiblast progenitors in the epiblast and Foxa2^high^ transitory progenitors that ingressed distally to the APS (Fig. 1a,b,a′,b′ and Extended Data Fig. 1a–d)^11^. Notably, the EMT transcription factor Snail1 was highly upregulated in T^+^ mesoderm progenitors, while it was weakly expressed on the mRNA level in Foxa2^high^ transitory progenitors (Figs. 1b,b′ and Fig. 2e and Extended Data Fig. 1d) and not expressed in mature Foxa2^high^/Sox17^+^ definitive endoderm (Extended Data Fig. 1e,g,h). During the transition to a mesenchymal state, Snail1 downregulates E-cadherin^17^. At the same time, N-cadherin is upregulated^20^. Immunostaining analysis revealed that mesodermal cells within the primitive streak displayed the well-described switch from E- to N-cadherin during EMT (Fig. 1c,c′ and Extended Data Fig. 1f). In contrast, FVF^high^ transitory progenitors, definitive endoderm and AME cells maintained E-cadherin and synchronously upregulated N-cadherin, as quantified by western blot and immunostaining (Fig. 1c–f and Extended Data Fig. 1f,i).

**Extended Data Fig. 2:**
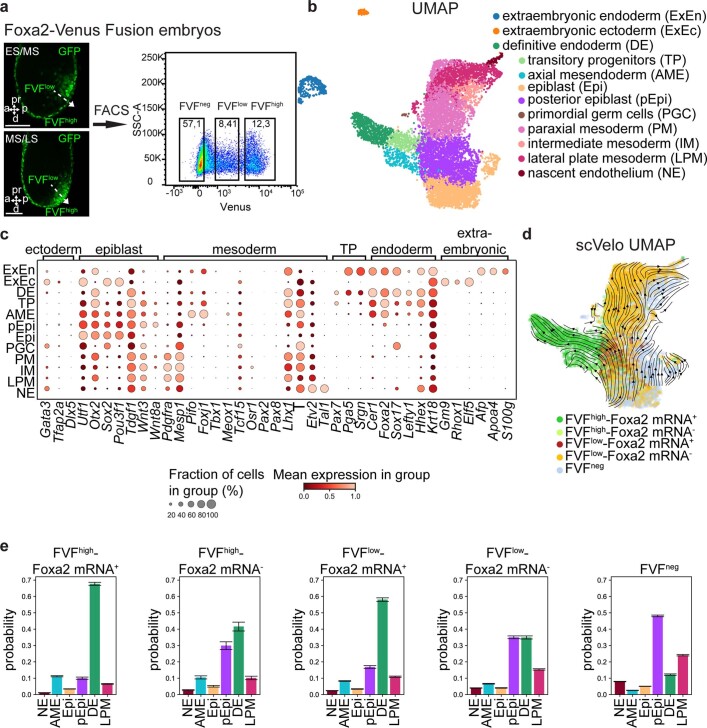
Mapping and single cell transcriptional profiling of gastrulation. **a**, Scheme for FACS sorting of FVF embryos for scRNA-seq analysis (2 FVF^neg^, 3 FVF^low^ and 3 FVF^high^ samples were used in the scRNAseq analysis). **b**, UMAP plot colored by cell population. **c**, Dotplot of tissue-specific marker genes (top brackets). Colors indicate expression level, dot size indicates fraction of cells expressing the gene. **d**, UMAP plot with RNA velocity arrows, colored by both FVF-sorting and presence/absence of Foxa2 mRNA. RNA velocity shows gene dynamics derived from abundance of unspliced and spliced mRNA molecules for each gene. **e**, Barplots of the fate probability for the FVF-sorted and Foxa2 mRNA positive or negative subpopulations of the pEpi (cell numbers from left to right, n = 308; n = 52; n = 287; n = 428; n = 1140). Bar height denotes mean fate probability per state and upper/lower whiskers indicate SEM. The higher a bar, the more likely is the indicated fate for this group of cells. All shown confocal images are single planes of a z-stack unless otherwise stated. Scale bar: 50 µm.

To understand the lineage bifurcations and hierarchy during gastrulation and to generate a continuous in vivo roadmap of the molecular changes, we combined FVF lineage labelling and flow sorting to perform high-throughput single-cell RNA sequencing (scRNA-seq) (Fig. 2a and Extended Data Fig. 2). This allowed us to enrich for rare transitory cell types and to map the FVF^low^ epiblast progenitors, FVF^high^ transitory progenitors and FVF^high^ AME and definitive endoderm descendants via Louvain cluster annotation (Extended Data Fig. 2a–d). Using our previously established scVelo^31^ and CellRank^32^ algorithms, we combined directional information from the RNA velocity and robustness of trajectory inference to compute fate probabilities, and identified lineage driver genes during mesoderm and endoderm segregation (Fig. 2b–d and Extended Data Fig. 2e). Combining FVF lineage labelling with pseudotime analysis revealed that, during posterior epiblast-to-endoderm transition, EMT transcription factor genes (*Snail1* and *2*, *Zeb1* and *2*, *Mesp1* and 2 and *Twist1*) are downregulated, whereas *E-cadherin* (*Cdh1*) is maintained and *N-cadherin* (*Cdh2*) is upregulated (Fig. 2d–f). In contrast, posterior epiblast cells undergoing a mesoderm transition upregulate an EMT transcription factor program and show an *E-* to *N-cadherin* switch during mesenchymal transition. Altogether, these results suggest that, in mouse embryos, Foxa2^low^ epiblast progenitors upregulate Foxa2 levels and give rise to Foxa2^high^ transitory progenitors that ingress distal to the anatomical visible primitive streak to form the Foxa2^high^/Sox17^+^ definitive endoderm independent of a full EMT–MET cycle.

### In vitro-generated definitive endoderm forms by partial EMT

**Extended Data Fig. 3:**
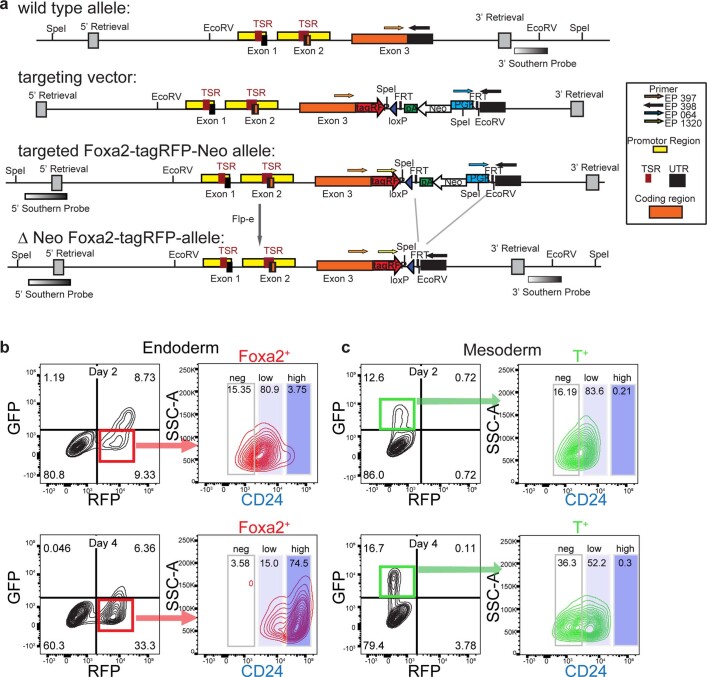
Characterization of endoderm and mesoderm *in vitro*. **a**, Targeting strategy to generate T^GFP/+^; Foxa2^tagRFP/+^ mESC line. An available T-GFP knock-in mESCs line^62^ was targeted with the described Foxa2^tagRFP^ construct. Transcriptional start region (TSR), untranslated region (UTR). **b**, Gating strategy of differentiated T^GFP/+^; Foxa2^tagRFP/+^ mESC line towards Foxa2^+^ (RFP^+^) endodermal cells and (**c**) T^+^ (GFP^+^) mesodermal cells. Differentiated cells were stained for CD24 and sorted according to their CD24 expression levels Foxa2^+^/CD24^low^, Foxa2^+^/CD24^high^, T^+^/CD24^low^ and T^+^/CD24^neg^ at day 2 and 4 of differentiation for global mRNA expression profile analysis.

**Extended Data Fig. 4:**
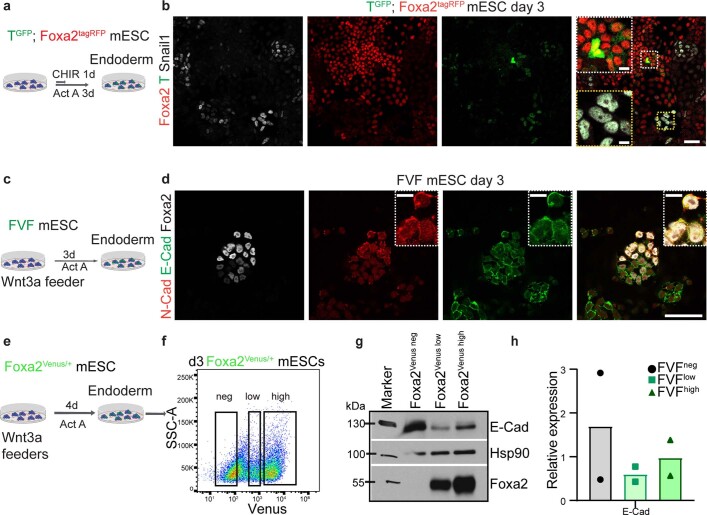
Endoderm does not show hallmarks of EMT *in vitro*. **a**, Endoderm differentiation scheme of T^GFP/+^; Foxa2^tagRFP/+^ mESC line. **b**, Confocal images of differentiated T^GFP/+^; Foxa2^tagRFP/+^ mESC line (representative of 3 independent differentiations) at day 3 stained for GFP (T), Foxa2 and Snail1. Note, the presence of T^+^, Snail1^+^ MES population (yellow box), Foxa2^high^/T^+^ AME progenitors (white box) as well as Foxa2^low^/^high^ DE progenitors, recapitulating the observations *in vivo*. **c**, Differentiation scheme of FVF mESCs towards endoderm fate. **d**, Confocal images of differentiated FVF mESCs (representative of 2 independent differentiations) at day 3 stained for Venus (Foxa2, white), E-cadherin and N-cadherin. **e**, Scheme of endoderm differentiation. **f**, Representative FACS plot of Foxa2^Venus/+^ cells after 3 days of differentiation, followed by FACS sorting of Foxa2^Venus neg^, Foxa2^Venus low^, Foxa2^Venus high^ for (**g**) western blot analysis (2 experiments) and (**h**) quantification of E-cadherin (n = 2 experiments). All samples are derived from biologically independent experiments. Data are presented as mean values. All shown confocal images are single planes of a z-stack unless otherwise stated. Scale bar: 50 µm, insets 10 µm. Source data

To verify these results in vitro and simultaneously analyse mesoderm and endoderm segregation, we generated a T^GFP/+^; Foxa2^tagRFP/+^ knock-in dual-reporter mESC line (Extended Data Fig. 3a). We differentiated the mESCs in a stepwise time-resolved manner into mesoderm and endoderm (Fig. 2g and Methods) and sorted progenitors and definitive lineages by flow sorting using the reporter (T^GFP^ and Foxa2^tagRFP^) and differential marker (CD24^neg/low/high^) expression (Extended Data Fig. 3b,c)^33^. Global transcriptional profiling at days 2 and 4 of differentiation revealed that mesoderm progenitors still express pluripotency markers (*Pou5f1*, *Nanog* and *Sox2*) and slightly upregulate mature mesoderm markers (*Tbx3* and *5*, *Mesp1* and *2* and *Hand1*) (Fig. 2h). Similarly, DEPs still express pluripotency genes and already induce the expression of mature definitive endoderm markers (*Sox17*, *Hhex*, *Dkk1* and *Cer1*) (Fig. 2h). Next, we tested whether EMT transcription factors are expressed during mesodermal and endodermal lineage acquisition in vitro. During mesoderm differentiation, EMT transcription factor genes (*Zeb1* and *2*, *Mesp1* and *2* and *Snail1*) are already expressed in mesoderm progenitors and further upregulated in mesoderm, while DEP and definitive endoderm do not upregulate EMT transcription factor genes (Fig. 2h). In vitro endoderm differentiations confirmed the absence of Snail1 protein in DEPs and definitive endoderm (Extended Data Fig. 4a,b and Methods). Furthermore, *E-cadherin* and *N-cadherin* mRNA and protein were maintained or upregulated, respectively, during endoderm differentiation in vitro (Fig. 2h and Extended Data Fig. 4c,d), consistent with our in vivo results (Fig. 1c–f). Using a previously generated knock-in Foxa2–H2B–Venus (Foxa2^Venus/+^) mESC transcriptional reporter line^34^, we flow sorted Foxa2^Venus low^ progenitor and Foxa2^Venus high^ definitive endoderm, which maintain the epithelial marker protein E-cadherin during differentiation (Extended Data Fig. 4e–h). Re-analysis of our previously generated RNA-seq, chromatin immunoprecipitation sequencing (ChIP-seq) data from stem cell-derived endoderm^34^ suggested that the expression of *N-cadherin* as well as *E-cadherin* is regulated by Foxa2 (Fig. 2h and Supplementary Table 1). Taken together, nascent endoderm expresses epithelial markers from specification to determination in vitro and in vivo. Furthermore, definitive endoderm is formed independent of an E- to N-cadherin switch and substantial upregulation of well-known EMT transcription factors, suggesting it is formed by partial EMT.

### The EMT transcription factor Snail1 is not required for endoderm formation

**Fig. 3:**
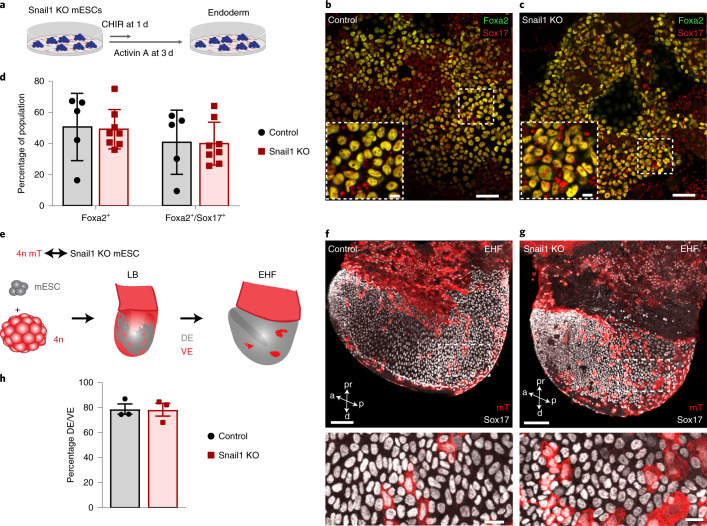
Snail1 is not required for endoderm formation. **a**, Endoderm differentiation schematic of Snail1 knockout mESCs. **b**,**c**, Immunohistochemistry of differentiated wild-type (control) (**b**) and Snail1 knockout (**c**) endoderm cells at day 3 stained for Sox17 and Foxa2. **d**, FACS quantification of Foxa2^+^ and Sox17^+^/Foxa2^+^ cells in control versus Snail1 knockout endoderm differentiations (two-tailed unpaired Student’s *t*-test (no significant difference); *n* = 5 (control); *n* = 8 (Snail1 knockout)). **e**, Schematic of the generation of tetraploid aggregation chimeras with Snail1 knockout mESCs. EHF, early headfold stage. LB, late bud stage. 4n, tetraploid. **f**,**g**, Maximum projection from confocal images of wild-type (control) (**f**) and Snail1 knockout chimeric embryos (**g**) stained for RFP (mT) and Sox17, showing the dispersal of visceral endoderm (mT^+^) by Snail1 mutant or wild-type definitive endoderm cells. The bottom images show magnified views of the areas highlighted by dashed white rectangles in the images above. **h**, Quantification of formed definitive endoderm over visceral endoderm in wild-type (control) and Snail1 mutant embryos at the early headfold stage (two-sided unpaired Student’s *t*-test (no significant difference); *n* = 3 embryos each). All samples were derived from biologically independent experiments. The images in **b** and **c** are representative of five independent differentiations each. The images in **f** and **g** are representative of three embryos each. The data are presented as mean values ± s.e.m. Scale bars, 50 µm (insets, 10 µm). Source data

**Extended Data Fig. 5:**
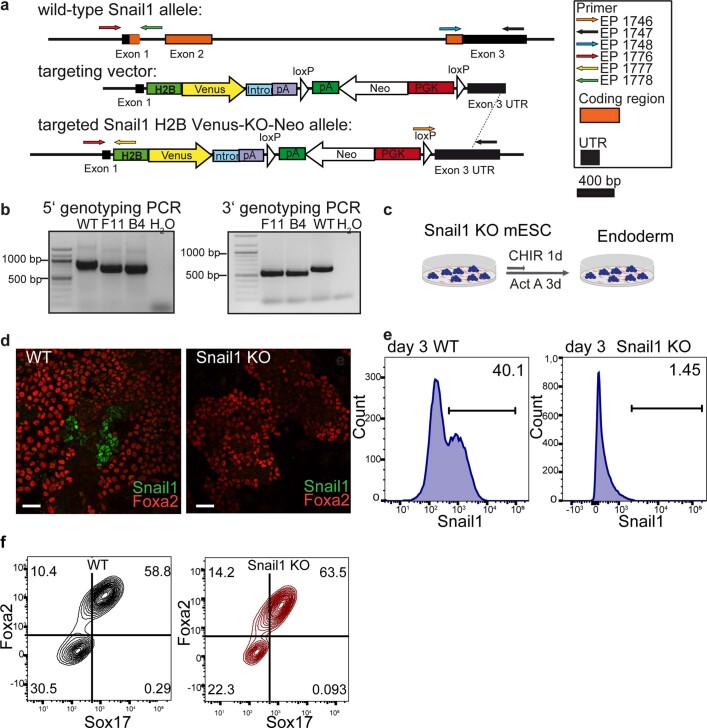
Generation of Snail1 knockout mESCs. **a**, Targeting strategy to generate Snail1 KO mESCs. **b**, 3′ end genotyping PCR (WT = 601 bp, KI = 529 bp) and 5′ end genotyping PCR (WT = 738 bp, KI = 657 bp) of the used Snail1 KO clones F11 and B4. **c**, Endoderm differentiation scheme of Snail1 KO mESCs. **d**, Confocal images of differentiated WT and Snail1 KO mESCs (representative of 2 independent differentiations) at day 3 stained against Snail1 and Foxa2. **e**, FACS analysis of differentiated WT and Snail1 KO mESCs (2 independent differentiations) at day 3 stained for Snail1 confirming the absence of Snail1 in the Snail1 KO mESCs line. **f**, FACS analysis of differentiated wildtype and Snail1 KO mESCs for Foxa2 and Sox17 expression (n = 5 (wildtype), n = 8 (Snail1 KO)). All samples are derived from biologically independent experiments. All shown confocal images are single planes of a z-stack unless otherwise stated. Scale bar: 50 µm, insets 10 µm. Source data

Several knockout studies have indicated redundant functions of the EMT transcription factors Zeb1 and 2 (refs. ^35,36^) and Snail2 (ref. ^37^) during gastrulation, whereas mutant embryos with alterations in Snail1 fail to downregulate E-cadherin and show defective EMT and mesoderm formation^17,18^. To test whether Snail1 function is required for definitive endoderm formation, we generated a Snail1 knockout mESC line (Extended Data Fig. 5a,b). After 3 d of definitive endoderm differentiation, we detected comparable numbers of definitive endoderm cells (Foxa2^high^/Sox17^+^) formed from both wild-type (control) and Snail1 knockout mESCs (Fig. 3a–d and Extended Data Fig. 5c–f). To analyse definitive endoderm formation in vivo, we generated completely mESC-derived embryos by tetraploid aggregation (Fig. 3e–g)^38^. This allowed us to observe nascent definitive endoderm formation (derived from mESCs) and visceral endoderm dispersal (derived from tetraploid embryos expressing membrane Tomato (mT)^39^) by means of fluorescent marker gene expression. At the early headfold stage, we observed that the visceral endoderm (mT^+^) was clearly dispersed by Snail1 knockout mESC-derived definitive endoderm (Sox17^+^), comparable to control chimeras (Fig. 3e–g). Together, these findings demonstrate that the master EMT transcription factor Snail1 is not necessary for definitive endoderm formation in vitro and in vivo.

### Foxa2 suppresses a complete EMT during endoderm formation

**Fig. 4:**
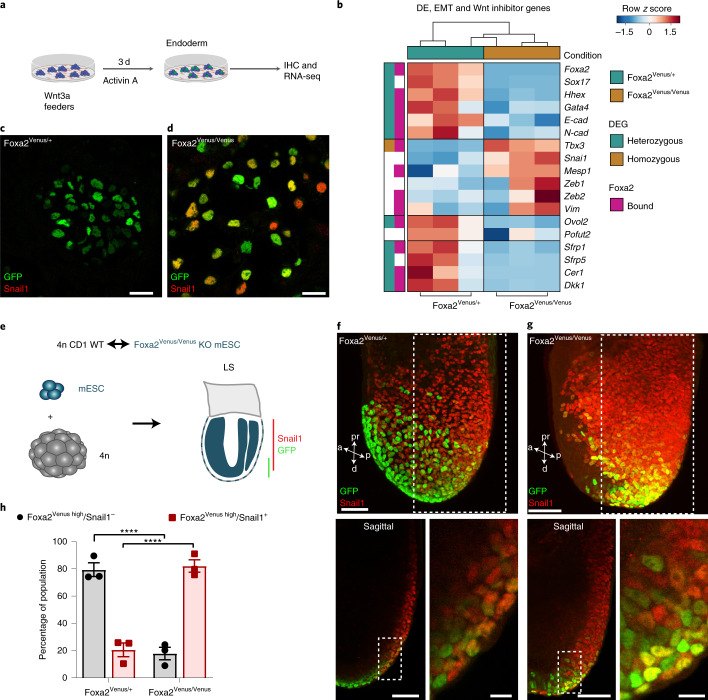
Foxa2 suppresses Snail1 in the endoderm lineage. **a**, Schematic of endoderm differentiation of Foxa2^Venus/Venus^ knockout mESCs. IHC, immunohistochemistry. **b**, Heatmap of Foxa2^Venus/+^ and Foxa2^Venus/Venus^ endoderm^34^ showing the up- and downregulation of endoderm genes, EMT activators, inhibitors and Wnt signalling inhibitors (*n* = 3 independent differentiations). The pink boxes indicate genes bound by Foxa2. The green and brown boxes mark genes with significantly different expression in Foxa2^Venus/+^ and Foxa2^Venus/Venus^ cells, respectively. **c**,**d**, Immunostainings against Venus and Snail1 of Foxa2^Venus/+^ control (**c**) and Foxa2^Venus/Venus^ knockout mESCs (**d**) differentiated under endoderm conditions for 3 d. **e**, Schematic of aggregation chimeras generated with Foxa2^Venus/+^ and Foxa2^Venus/Venus^ mESCs. LS, late-streak stage; WT, wild type. **f**,**g**, Maximum projections (top) and single optical sections (bottom) from confocal images of Foxa2^Venus/+^ (**f**) and Foxa2^Venus/Venus^ late-streak stage mESC tetraploid aggregation chimeras (**g**) stained for Venus and Snail1. **h**, Quantification of Venus^high^ and Snail1^+^ or Snail1^–^ cells in Foxa2^Venus/+^ and Foxa2^Venus/Venus^ aggregation chimeras (****P < 0.0001; ordinary one-way analysis of variance with Tukey’s multiple comparisons test; *n* = 3 embryos). Statistically non-significant results are not indicated in the figure. All samples were derived from biologically independent experiments. The images in **c** and **d** are representative of four independent endoderm differentiations. The images in **f** and **g** are representative of three embryos each. The data are presented as mean values ± s.e.m. Scale bars, 50 µm (insets, 10 µm). Source data

To better understand how definitive endoderm is formed during gastrulation, we investigated the role of Foxa2, which has been shown to be crucially important for the formation of epithelial lineages (that is, ADE and AME)^9,10^. For this purpose, we used our previously established knock-in/knockout Foxa2 reporter line for endoderm differentiations and re-analysed our RNA-seq and ChIP-seq datasets^34^. As predicted, we observed a lack of definitive endoderm formation and mature marker expression using the homozygous Foxa2^Venus/Venus^ compared with heterozygous Foxa2^Venus/+^ definitive endoderm cells (Fig. 4a,b). This resembled the well-known phenotype of Foxa2 mutant embryos^9–11,35^. Interestingly, we noted that, upon loss of Foxa2, EMT transcription factor genes (*Snail1*, *Mesp1*, *Tbx3* and *Zeb1* and *2*) and the EMT marker gene (*Vim*) were upregulated in Foxa2^Venus/Venus^ mutant cells (Fig. 4b–d). To analyse whether Snail1 is also upregulated in the Foxa2 lineage in vivo, we generated mESC-derived embryos using heterozygous Foxa2^Venus/+^ and homozygous Foxa2^Venus/Venus^ knock-in/knockout mESCs by tetraploid complementation (Fig. 4e–h). At the late-streak stage, the APS region was thickened due to an accumulation of Foxa2 lineage-positive cells in the Foxa2^Venus/Venus^ mESC-derived embryo (compare Fig. 4f,g)^11^. We also noticed more Venus lineage-labelled Foxa2 mutant cells with clear upregulation of the EMT transcription factor Snail1 (Fig. 4f–h). Induction of Snail1 expression in the Foxa2^Venus/Venus^ mutant lineage-labelled cells in vitro as well as in vivo implied that Foxa2 suppresses the key EMT transcription factor Snail1 to prevent E-cadherin downregulation and mesenchymal transition.

### Foxa2 serves as an epithelial gatekeeper in the endoderm

**Fig. 5:**
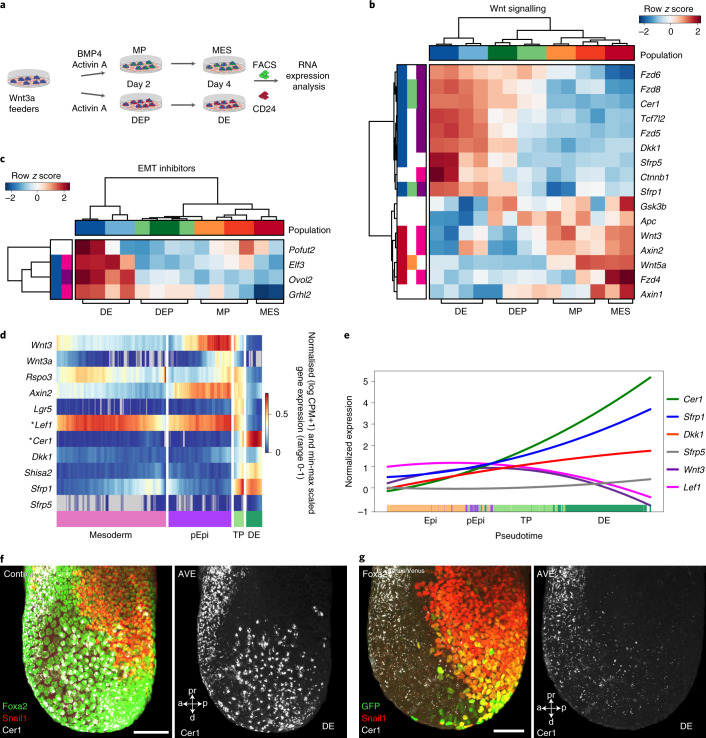
Foxa2 activates Wnt inhibitors in endoderm. **a**, Schematic of endoderm and mesoderm differentiation of T^GFP/+^; Foxa2^tagRFP/+^ mESCs. **b**, Heatmap of FACS-sorted endodermal and mesodermal subpopulations expressing different levels of CD24 at days 2 and 4, showing RNA expression levels of canonical Wnt signalling genes. **c**, Heatmap of endoderm and mesoderm from differentiations of T^GFP/+^; Foxa2^tagRFP/+^ cells, showing upregulation of EMT suppressors in endoderm. In **b** and **c**, the coloured boxes to the left indicate genes that were differentially expressed in DEP versus mesoderm progenitor (green and orange, respectively) or definitive endoderm versus mesoderm (blue and red, respectively) and whether Foxa2 binds (pink) or binds and regulates them (purple). **d**, Clustered heatmap showing the smoothed (sliding window of *n* = 100 cells) and scaled gene expression of Wnt signalling genes in mesoderm (paraxial mesoderm, intermediate mesoderm, LPM and nascent endothelium), posterior epiblast, transitory progenitors and definitive endoderm. **e**, Quadratic spline plots showing genes involved in activation and inhibition of Wnt signalling along diffusion pseudotime from epiblasts to posterior epiblasts to transitory progenitors to definitive endoderm. **f**,**g**, Maximum projections of control (representative of six embryos) (**f**) and Foxa2^Venus/Venus^ knockout (representative of eight embryos) (**g**) late-streak-stage aggregation embryos immunostained for GFP or Foxa2, Snail1 and Cer1. All samples were derived from biologically independent experiments. All shown confocal images are single planes of a z stack unless otherwise stated. Scale bars, 50 µm.

**Fig. 6:**
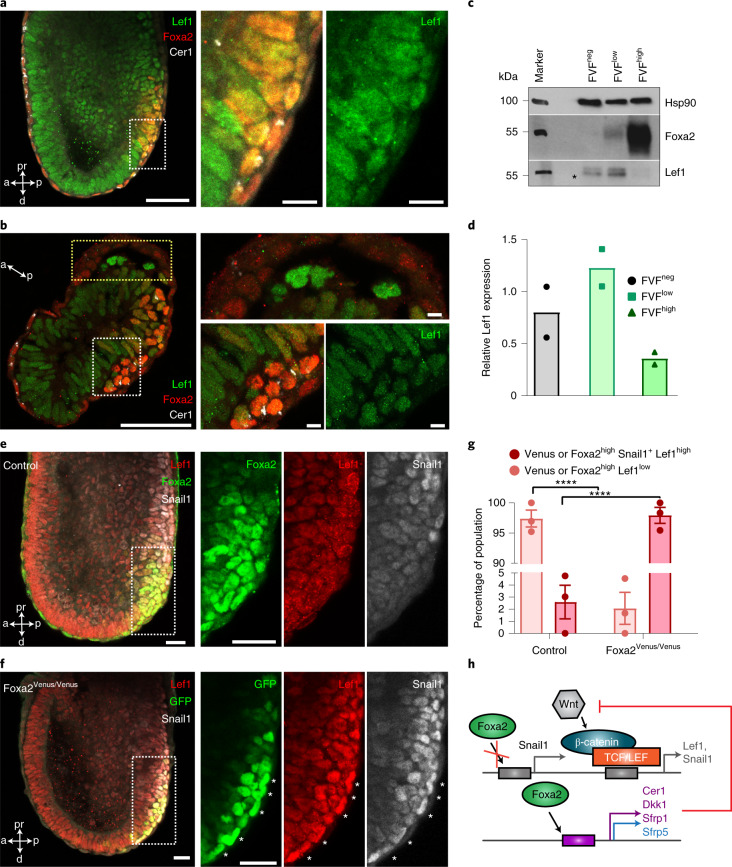
Foxa2 indirectly suppresses Wnt signalling in endoderm. **a**,**b**, Confocal image of a mid-streak-stage embryo (**a**) and a transverse section through the epiblast of a mid-streak-stage embryo (**b**) immunostained for Foxa2, Lef1 and Cer1. **c**,**d**, Western blot analysis (**c**) and quantification (**d**) of Lef1 from FACS-sorted FVF^neg^, FVF^low^ and FVF^high^ cells of *n* = 36 and *n* = 122 FVF embryos (*n* = 2 experiments). The asterisk marks unspecific bands. **e**,**f**, Representative confocal images of control (**e**) and Foxa2^Venus/Venus^ knockout late-streak-stage aggregation chimeras (**f**) immunostained for Foxa2/GFP, Lef1 and Snail1. **g**, Quantification of Venus or Foxa2^high^ cells colocalizing with either Snail1 and Lef1^high^ or Lef1^low^ in control versus Foxa2 knockout aggregation embryos (****P < 0.0001; ordinary one-way analysis of variance with Tukey’s multiple comparison test; *n* = 3 embryos). The data are presented as mean values ± s.e.m. Statistically non-significant results are not indicated in the figure. **h**, Schematic illustrating how Foxa2 inhibits a full EMT in endoderm. Foxa2 directly (purple) or indirectly (blue) controls the expression of Wnt inhibitors. The purple and grey boxes represent the transcription factor-binding sites of gene specific promoters. TCF/LEF, T-cell factor/lymphoid enhancer factor. All samples were derived from biologically independent experiments. The images in **a**,**b**,**e** and **f** are representative of six, three, three and three embryos, respectively. All shown confocal images are single planes of a z stack unless otherwise stated. Scale bars, 50 µm (insets, 10 µm). Source data

TGF-β and nodal and canonical Wnt/β-catenin signalling activates EMT transcription factors and initiates gastrulation^40–42^. Recently, it was shown in different cancer and epithelial cell lines that the EMT key regulator Snail1 is induced by the TGF-β ligand 1 (ref. ^43^) and Wnt/β-catenin activation by Gsk3β inhibition^37,44,45^. In FVF^high^ transitory progenitors and definitive endoderm, we did not observe substantial upregulation of Snail1 expression and synthesis (Figs. 1 and 2); however, in homozygous Foxa2^Venus/Venus^ mutant cells, Snail1 was highly expressed (Fig. 4c–h), suggesting that Foxa2 is a potential repressor of Snail1. However, ChIP-seq analysis of definitive endoderm cells revealed that *Snail1* *cis*-regulatory elements are not directly bound by Foxa2 (Fig. 2h and Supplementary Table 1)^34^. Next, we analysed the activity of Wnt signalling genes and observed that canonical Wnt ligands and its targets, such as *Wnt3* and *APC*, are not upregulated in definitive endoderm, in contrast with T^+^ mesoderm, which highly expresses these genes (Fig. 5a,b). Interestingly, secreted inhibitors of the canonical Wnt/β-catenin pathway (*Cer1*, *Dkk1* and *Srfp1* and *5*) are highly upregulated in definitive endoderm but downregulated in Foxa2^Venus/Venus^ knockout cells (Figs. 4b and 5a,b). Further analysis of the ChIP-seq data suggested that several of the secreted inhibitors of the canonical Wnt/β-catenin pathway are directly regulated by Foxa2, such as Cer1 (Fig. 5b and Supplementary Table 1). During gastrulation, we observed downregulation of Wnt ligands (*Wnt3* and *Wnt3a*) and the target gene (*Lef1*), as well as upregulation of secreted Wnt inhibitors (*Cer1*, *Dkk1* and *Sfrp1* and *5*) in the transition from the posterior epiblast to transitory progenitors and definitive endoderm (Fig. 5d,e). Interestingly, the AVE and ADE marker protein Cer1 is already upregulated in Foxa2^high^ transitory progenitors and definitive endoderm at the posterior side of gastrula embryos (Figs. 5f and 6a,b), which correlates with the downregulation of the Wnt target gene and transcription factor Lef1 (Fig. 6a–d). In line with our Foxa2 knockout results from in vitro differentiations (Fig. 4b), we also observed a lack of Cer1 expression in Venus^+^ lineage-labelled cells of Foxa2^Venus/Venus^ aggregation chimeras (Fig. 5g) and concomitant upregulation of the Wnt target gene *Lef1* and the EMT transcription factor Snail1 (Fig. 6e–g). These results suggest that Foxa2 directly induces the expression of Wnt inhibitors and thereby indirectly inhibits Wnt/β-catenin signalling and target genes, such as *Lef1* and *Snail1* (Fig. 6h)^37,44–47^.

**Extended Data Fig. 6:**
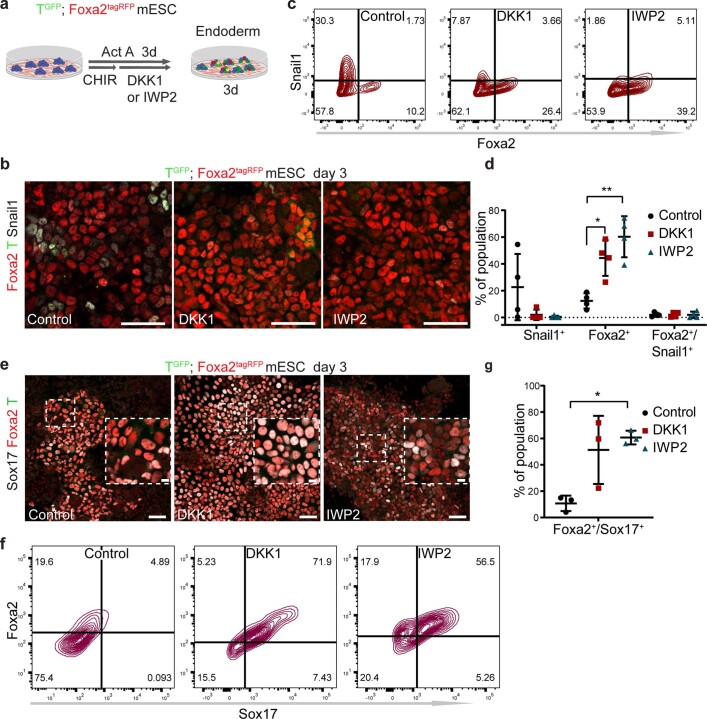
Inhibition of Wnt signaling promotes endoderm differentiation. **a**, Scheme of endoderm differentiation under normal conditions (control) and with the supplementation of DKK1 or IWP2. **b**, Immunohistochemistry (representative of 3 independent differentiations), (**c**) FACS analysis and (**d**) quantification of differentiated T^GFP/+^; Foxa2^tagRFP/+^ under control, DKK1 and IWP2 conditions stained for GFP (T), Foxa2 and Snail1 (Ordinary one-way ANOVA with Bonferroni’s multiple comparison test, n = 4 independent differentiations). **e**, Confocal images (representative of 3 independent differentiations), (**f**) FACS analysis and (**g**) quantification of T^GFP/+^; Foxa2^tagRFP/+^ mESC differentiated for 3 days under control, DKK1 or IWP2 conditions and stained for GFP (T), Foxa2 and Sox17 (Ordinary one-way ANOVA with Bonferroni’s multiple comparison test, n = 3 independent differentiations). All samples are derived from biologically independent experiments. Data are presented as mean values ±SEM. **d**, *P < 0.0146, **P < 0.0011, (**g**) *P < 0.0233. Statistically non-significant results are not indicated in the figure. All shown confocal images are single planes of a z-stack unless otherwise stated. Scale bar: 50 µm, insets 10 µm. Source data

To test whether inhibition of canonical Wnt signalling represses Snail1 expression and promotes epithelial endoderm formation, we differentiated T^GFP/+^; Foxa2^tagRFP/+^ mESCs for 1 d with addition of the Gsk3β inhibitor CHIR and activin A to induce endoderm formation (Extended Data Fig. 6a–d). Next, we supplemented the medium with the Wnt inhibitor Dickkopf 1 (DKK1) or the Wnt ligand secretion inhibitor IWP2 for the following 2 d. The cells treated with Wnt inhibitors showed decreased Snail1 expression compared with control cells, which was expected and confirmed that Snail1 is a Wnt/β-catenin target gene^37,44,45^. Interestingly, the Foxa2^+^/Sox17^+^ definitive endoderm population was increased from ~11 to ~51% in DKK1-supplemented cultures and ~60% in IWP2-supplemented cultures (Extended Data Fig. 6e–g). Altogether, these results show that Foxa2 activates Wnt/β-catenin inhibitors to indirectly repress *Snail1* expression to promote progenitor differentiation towards an epithelial endodermal fate in vitro and in vivo.

During endoderm formation, Foxa2 regulates a molecular program to induce and maintain endodermal polarization and epithelialization^11,48^. Consistently, we observed that FVF^high^ transitory progenitors and FVF^high^/Sox17^+^ definitive endoderm descendants do not undergo a complete EMT–MET cycle, but instead acquire epithelial cell plasticity to change their morphology from a columnar- to a squamous-shaped epithelium (Figs. 1 and 2, Extended Data Fig. 1a and Supplementary Video 1)^11,29,30^. Interestingly, we found upregulation of several EMT suppressors, such as *GRHL2* (refs. ^49,50^), *Ovol2* (ref. ^51^), *Pofut2* (ref. ^52^) and *Elf3* (ref. ^53^) in endoderm compared with mesoderm (Fig. 5c). Furthermore, ChIP-seq data analysis suggested that *Ovol2* is bound and potentially regulated by Foxa2 (Fig. 5c and Supplementary Table 1). Analysis of RNA-seq data from Foxa2^Venus/+^ and Foxa2^Venus/Venus^ mESC differentiations revealed that these EMT inhibitors are downregulated upon loss of Foxa2 (Fig. 4b). Altogether, these findings show that Foxa2 not only counteracts a complete EMT in endoderm progenitors and the definitive endoderm lineage by the induction of Wnt inhibitors and EMT suppressors, but also quickly re-establishes an epithelial identity by the activation of target genes that regulate cell polarity and adhesion^11^. Thus, Foxa2 serves as an epithelial gatekeeper and EMT suppressor during endoderm formation.

### Dynamic molecular changes drive endoderm differentiation

**Fig. 7:**
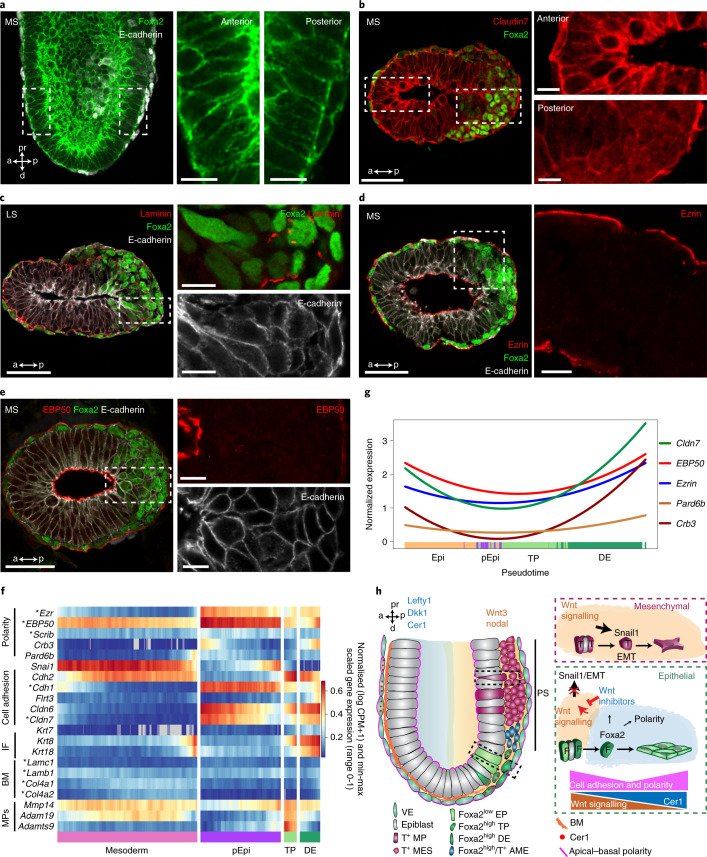
Epithelial cell plasticity drives endoderm formation. **a**, Representative image of a mid-streak-stage embryo stained for Foxa2 and E-cadherin. **b**–**e**, Transverse sections through the epiblasts of wild-type mid- and late-streak-stage embryos immunostained for Foxa2 and Claudin7 (**b**), Foxa2, laminin and E-cadherin (**c**), Foxa2, Ezrin and E-cadherin (maximum projection) (**d**) and Foxa2, EBP50 and E-cadherin (**e**). **f**, Clustered heatmap showing the smoothed (sliding window of *n* = 100 cells) and scaled gene expression of polarity, cell adhesion, intermediate filaments (IF), basement membrane (BM) and metalloproteinases (MPs) in mesoderm (paraxial mesoderm, intermediate mesoderm, LPM and nascent endothelium), posterior epiblasts, transitory progenitors and definitive endoderm. The asterisks mark genes that have been confirmed by immunohistochemistry. **g**, Quadratic spline plot showing the expression of polarity and cell adhesion genes along diffusion pseudotime from epiblast to posterior epiblast to transitory progenitors to definitive endoderm. **h**, Schematic of endoderm formation by partial EMT. All samples were derived from biologically independent experiments. The images in **a**–**d** are representative of three embryos each. All shown confocal images are single planes of a z stack unless otherwise stated. Scale bars, 50 µm (insets, 10 µm).

**Extended Data Fig. 7:**
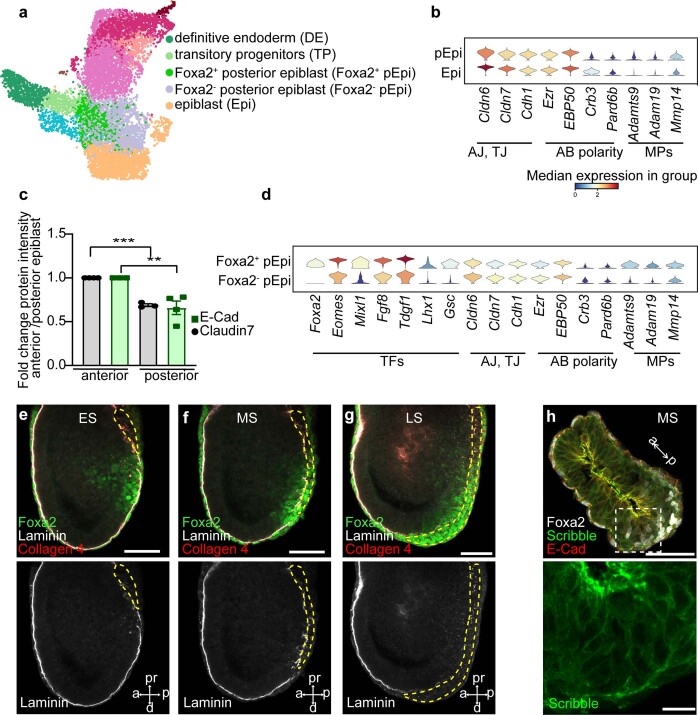
Epithelial cell plasticity drives endoderm formation. **a**, UMAP plot colored by cell population. **b**, Stacked violin plots showing the gene expression distribution (columns) of AJ, TJ, AB polarity and metalloproteinases (MPs) genes in pEpi (*n* = 2215) versus Epi (*n* = 1929). Color corresponds to normalized median gene expression for each group. **c**, Quantification of E-cadherin (one-way ANOVA, n = 4 embryos) and Claudin7 (one-way ANOVA, n = 3 embryos) expression intensity at the anterior and posterior epiblast of MS stage embryos. **d**, Stacked violin plots showing the expression of TFs, AJ, TJ, AB polarity and metalloproteinases (MPs) genes in Foxa2^+^ pEpi (*n* = 616) versus Foxa2^-^ pEpi (*n* = 1599). **e**, Immunostainings for Foxa2, Laminin and Collagen 4 of ES, (**f**) MS and (**g**) LS stage embryos (representative of 7 embryos used for (**e**-**g**). Yellow dashed line indicates PS. **h**, MS stage embryo (3 embryos) stained for Foxa2, Scribble and E-cadherin. All samples are derived from biologically independent experiments. Data are presented as mean values ±SEM.; **P < 0.0017, ***P < 0.0054. Statistically non-significant results are not indicated in the figure. All shown confocal images are single planes of a z-stack unless otherwise stated. Scale bar: 50 µm, insets 10 µm. Source data

Finally, to understand how the definitive endoderm is formed on the morphogenetic level, we analysed and verified our time-resolved and lineage-mapped scRNA-seq data. We first compared differences of the anterior versus posterior epiblast epithelium. This disclosed lower expression of apical–basal polarity (*Crb3*, *Ezr* and *EBP50*) and tight junction genes (*Cldn6* and *7*) (Extended Data Fig. 7a,b). Both adherens junction E-cadherin and the tight junction protein Claudin7 were less abundant in posterior epiblast cells, and Claudin7 appeared in the cytoplasm and less at the plasma membrane (Fig. 7a,b and Extended Data Fig. 7c). Next, we compared Foxa2^−^ with Foxa2^+^ posterior epiblast cells (Extended Data Fig. 7a,d). Foxa2^+^ posterior epiblast cells showed a distinct transcription factor gene expression profile (*Foxa2*, *Eomes*, *Mixl1*, *Lhx1* and *Gsc*), but also particularly high expression of metalloproteinase genes (*Adamts9*, *Adam19* and *Mmp14*) involved in basement membrane remodelling and morphogenetic dynamics during gastrulation^54^. Interestingly, the high expression of metalloproteinases was maintained in Foxa2^high^ transitory progenitors (Fig. 7) and correlated with basement membrane remodelling in Foxa2^+^ posterior epiblasts (Fig. 7 and Extended Data Fig. 7e–g). Interestingly, we observed a transient downregulation of apical–basal polarity (*Ezr*, *Crb3*, *EBP50*, *Pard6b* and *Scribble*) and tight junction genes (*Cldn6* and *7*) and proteins in the endoderm transition from Foxa2^low^ posterior epiblast to Foxa2^high^ transitory progenitors and definitive endoderm (Fig. 7 and Extended Data Fig. 7h), probably due to a lack of basement membrane polarity cues. Altogether, the transition of columnar Foxa2^low^ posterior epiblast through Foxa2^high^ transitory progenitors to squamous Foxa2^high^/Sox17^+^ definitive endoderm shows several attributes of an epithelial cell plasticity program, including simultaneous expression of E- and N-cadherin and transient downregulation of polarity and adherens junction and tight junction proteins, best defined by a partial EMT^20,55^.

## Discussion

Here, we present a revised concept of germ layer formation during gastrulation (Fig. 7h). Before gastrulation is initiated, T^+^ mesoderm progenitors and Foxa2^low^ epiblast progenitors are already specified in the posterior epiblast. Mesoderm progenitors ingress into the primitive streak by a complete EMT to commit to a mesenchymal fate. In contrast, Foxa2^+^ posterior epiblasts and Foxa2^high^ transitory progenitors leave the posterior epiblast epithelium by transient upregulation of metalloproteinases, remodelling of the basement membrane and a transient downregulation of apical–basal polarity genes and proteins—features reminiscent of a partial EMT program. During this process, we observed an absence of EMT transcription factors, maintained E-cadherin expression, activation of EMT suppressors and expression of adherens junction and tight junction genes and proteins^12,13,24–28^. The co-expression of E- and N-cadherin in the nascent definitive endoderm and visceral endoderm probably increases the segregation of the endoderm from the mesoderm germ layer by differential cell adhesion, as proposed by Townes and Holtfreter in 1955^56^. Previously, we have shown that Foxa2^high^ transitory progenitors re-establish apical–basal polarity, adherens junctions and tight junctions while they start to express Sox17 and intercalate into the overlying visceral endoderm layer^11,29,30^.

Importantly, Foxa2 acts as an epithelial gatekeeper and EMT suppressor to protect endoderm progenitors from undergoing a complete mesenchymal transition. Foxa2 is necessary to prevent EMT transcription factor activation via the activation of canonical Wnt/β-catenin signalling inhibitors. Thus, these results are not only important to understand basic mechanisms of gastrulation, but also have broader implications, as EMT causes detrimental cancer metastasis. Importantly, endoderm-derived lung, pancreatic and colorectal cancers are among the most common and deadliest cancers worldwide (National Cancer Institute). So far, cancer metastasis was always associated with EMT but recent studies contradict the necessity of a complete EMT–MET cycle for the dissemination of cancer cells and for invasion and metastasis in pancreatic cancer^55,57,58^. Mechanisms of epithelial cell plasticity might allow cancer cell dissemination and metastasis and require further in-depth mechanistic studies to provide alternative targets for therapeutic intervention. In line with our basic developmental biology findings, it was shown that Foxa2 antagonizes a full EMT process in pancreatic and lung cancer^59–61^. Foxa2 is an EMT suppressor but also an epithelial gatekeeper; thus, maintaining FOXA2 expression during cancer initiation and progression will prevent a complete EMT–MET cycle and eventually cancer dissemination.

## Methods

### Mouse strains

FVF^30^, SCF × FVF^29^, mT/mG^39^ and CD-1 mice were kept and experiments were performed at the central facilities at the Helmholtz Zentrum München German Research Center for Environmental Health in accordance with German animal welfare legislation and acknowledged guidelines of the Society for Laboratory Animal Science and Federation of European Laboratory Animal Science Associations. Mice were kept under specific pathogen-free conditions in animal rooms with a light/dark cycle of 12 h/12 h, a temperature of 20–24 °C and a humidity of 45–65%. Mice received sterile filtered water and a standard diet for rodents ad libitum. For embryo generation, females at the age of ≥6 weeks and males at the age of ≥8 weeks were used.

### Cell lines

The cell lines used during this study were as follows: T^GFP/+^;Foxa2^tagRFP/+^ (ref. ^62^) G4 mESCs, FVF IDG3.2 mESCs^30^, IDG3.2 mESCs^63^, Snail1 knockout IDG3.2 mESCs and Foxa2–H2B–Venus IDG3.2 mESCs^34^.

### Gene targeting

The strategies for targeting T^GFP/+^; Foxa2^tagRFP/+^ and Snail1 knockout constructs are outlined in Extended Data Figs. 3a and 5. To generate the T^GFP/+^; Foxa2^tagRFP/+^ dual-reporter mESC line, we generated a targeting vector where the open reading frame (ORF) of Foxa2 in exon 3 was fused to the ORF of the red fluorescent protein (RFP) tagRFP, followed by a phospho-glycerate kinase promoter-driven Neomycin (Neo) resistance gene cassette flanked by two loxP sites. The plasmid construct was introduced by electroporation into an available T-GFP knock-in mESCs line, in which the Brachyury expression was disrupted by insertion of a green fluorescent protein (GFP) mini gene^62^. Neo-resistant clones were analysed by Southern blot and PCR with the primers EP064, EP397, EP398 and EP1320 (Supplementary Table 2).

To generate Snail1 knockout mESCs, we designed a targeting vector that replaced the Snail1 ORF by an H2B–Venus–Intron–polyA Neo cassette^64^. Two guide RNAs (gRNAs) were designed to cut around the start codon of Snail1 (gRNAs 101 and 129) and a further two gRNAs, used to cut around the stop codon of Snail1 (gRNAs 4 and 7), were cloned into the pbs–U6 vector. mESCs were transfected with the Snail1 knockout targeting vector, four gRNAs and Cas9–D10A overexpression vector (pCAG–Cas9–D10A–bpA). At 48 h after transfection, cells were selected with G418 and picked clones were analysed by PCR genotyping (Extended Data Fig. 5 and Supplementary Table 2). The recombination borders of the targeting vector and wild-type sequence were analysed by sequencing of the knock-in-specific PCR product.

### Aggregation chimeras

Aggregation chimeras were generated as described by Artus and Hadjantonakis^38^.

### Immunofluorescence stainings of whole-mount embryos

Embryos (E6.5–7.5) were dissected in Dulbecco’s phosphate-buffered saline (DPBS) and immediately fixed for 20 min with 2% paraformaldehyde (PFA) in DPBS at room temperature while shaking. The fixation was stopped by rinsing the embryos 2× with DPBS containing 0.1% Tween 20 (Merck; P9416) (DPBST). The embryos were permeabilized for 10 min (≤E7.5) to 15 min (>E7.5) using 0.1 M glycine (Merck; G8898) and 0.1% Triton X-100 (Merck; 108643) in Milli-Q water, then rinsed 2× with DPBST. Unspecific antibody binding was prevented by incubating the embryos in blocking solution containing 0.1% Tween 20, 10% heat-inactivated foetal calf serum (FCS), 0.1% bovine serum albumin (BSA) and 3% donkey serum in DPBST for ≥1 h at room temperature while shaking. Subsequently, the primary antibodies diluted in blocking solution (for dilutions, see the antibody list in Supplementary Table 3) were added and the embryos were kept at 4 °C overnight on a shaker and for another 1–2 h at room temperature the following day. The embryos were rinsed 2× and washed 3× for 10 min with DPBST. Subsequently, the embryos were exposed to the secondary antibodies diluted in blocking solution for at least 3 h at room temperature on a shaker. The secondary antibodies were replaced by a 4′,6-diamidino-2-phenylindole (DAPI)/DPBST solution (2 µg ml^−1^ DAPI in DPBST) and incubated for 20 min at room temperature. Afterwards, the embryos were rinsed twice and then washed 3× for 10 min with DPBST. The embryos were dehydrated in 15 and 30% glycerol in DPBS, each for 10 min at room temperature. The embryos were then embedded in antifade between two cover slips using a 100-µm spacer, dried at room temperature and stored at 4 °C until imaging. A list of primary and secondary antibodies is shown in Supplementary Table 3.

### Paraffin immunohistochemistry

Embryos were fixed in 4% PFA overnight, embedded in HistoGel (Richard-Allan Scientific; HG-4000-144), dehydrated in alcohol gradients, embedded in wax blocks and sectioned at 6-µm thickness. Slides were cleared with xylene, rehydrated and permeabilized in 1% sodium dodecyl sulfate (SDS) before performing antigen retrieval (Diva Decloaker; Biocare Medical).

Blocking was done with 10% normal donkey serum and incubation was performed with primary antibodies overnight at 4 °C. Slides were then washed 3× with PBST and incubated with fluorophore-conjugated secondary antibodies for 1 h at room temperature in the dark. Nuclei were stained with DAPI (Life Technologies, 1:500–1:1,000). After washing, the slides were finally mounted with Vectashield (Vector Laboratories; H-1000-10) and imaged using a confocal microscope.

### Immunofluorescence stainings of cryosections from embryos

Embryos were fixed for 30 min to 1 h in 2% PFA, washed twice in PBS and dehydrated in 5, 10, 15, 20 and 30% sucrose (1 h each) and 30% sucrose/O.C.T. (Tissue-Tek; 4583) (1:1) overnight at 4 °C. Embryos were sectioned at 12- to 15-µm thickness and stored at −20 °C until usage. The sectioned embryos were permeabilized and blocked (see above). Primary and secondary antibody staining was performed as described above. Finally, the slides were mounted with Vectashield and kept for 24 h at room temperature to dry. Supplementary Table 3 lists the antibodies and dilutions used.

### Immunofluorescence stainings of cells

Differentiated cells were washed with PBS and fixed for 15 min with 4% PFA. The cells were immunostained as described in the previous section. The cells were kept in PBS for immediate imaging. Detailed information about the primary and secondary antibodies used is provided in Supplementary Table 3.

### Immunofluorescence stainings for flow cytometry analysis

Differentiated mESCs were dissociated using Accutase (Merck; A6964) or 0.05% trypsin (Life Technologies; 25300054) and fixed in 4% PFA for 10 min. The cells were permeabilized for 15 min and blocked for 1 h at room temperature (see above). Next, primary antibodies were diluted in blocking solution and incubated for 3–4 h at room temperature or overnight at 4 °C. Cells were washed 3× with PBS for 10 min each and secondary antibody solution was added for 1–2 h at room temperature. After another washing of 3× with PBS, samples were analysed by BD FACSAria III and FlowJo version 10.2. The gates were determined by secondary antibody controls. A list of all primary, secondary and conjugated antibodies is provided in Supplementary Table 3.

### Fluorescence-activated cell sorting (FACS) for scRNA-seq and western blotting

Embryos were isolated and extraembryonic compartments were removed mechanically. Embryonic compartments were washed with cold PBS. For single-cell suspension, FVF embryos and differentiated Foxa2^Venus/+^ mESCs were incubated with TrypLE (Life Technologies; 12605) for 10 min at 37 °C. Cells were dissociated by pipetting up and down. To exclude dead cells, samples were incubated for 5–10 min with either 7-aminoactinomycin D or DAPI on ice in the dark. The samples were then washed twice, resuspended in FACS buffer (PBS, 2% FCS and 2 mM ethylenediaminetetraacetic acid) and loaded for flow sorting. The gating strategy was as follows: main population > single cells > living cells (7-aminoactinomycin D/DAPI negative) > FVF^neg^, FVF^low^ and FVF^high^ cells. For scRNA-seq analysis, the cells were collected in RNA-seq buffer (PBS and 1% FCS). For western blotting analysis, the cells were collected in PBS and spun down and the pellet was snap frozen until it was used.

### Western blotting

For western blotting of FACS-sorted cells, the cell pellet was dissociated by RIPA buffer (75 mM NaCl, 6.37 mM sodium deoxycholate, 0.005% NP-40, 0.05% SDS and 25 mM Tris (pH 8)) supplemented with phosphatase inhibitor cocktail. The cell lysates were resolved by SDS–polyacrylamide gel electrophoresis, then transferred to a polyvinylidene fluoride membrane (Bio-Rad) and blocked in 5% BSA–TBST (TBS + 0.1% Tween 20) for 1 h. The primary antibodies were incubated in 5% BSA–TBST overnight at 4 °C on a shaker. The following day, the membranes were washed at least three times for 15 min in TBST on a shaker. The secondary horseradish peroxidase-conjugated antibodies were incubated with shaking for 1–2 h at room temperature. After washing the membranes with TBST three times for 15 min each, the bands were visualized by adding Pierce ECL Western Blotting Substrate (Thermo Fisher Scientific). The bands were quantified with ImageJ version 1.53c. Supplementary Table 3 lists the primary and secondary antibodies and their dilutions.

### Image analysis

Images from immunostained embryos were acquired with Leica SP5 and Zeiss LSM 880 Airyscan confocal microscopes. Images taken with the Leica confocal microscope were analysed using Leica LAS AF Lite 4.0 and images taken with the Zeiss confocal microscope were processed using Zeiss Zen 2.3 lite Blue software.

### Quantifications

Western blots were quantified with ImageJ version 1.53c by determining the pixel density of the protein of interest. After background subtraction, the ratio was determined by protein of interest/loading control.

For all of the quantifications of different cell populations, an entire confocal z stack of an embryo was analysed using ImageJ version 1.53c. For every staining, three individual embryos were processed in the same way. In brief, for Fig. 3h, Sox17^+^mT^−^ (definitive endoderm) cells and Sox17^+^mT^+^ (visceral endoderm) cells at the surface of the Snail1 variant and wild-type embryos were counted. For Fig. 4h, Venus^high^ and either Snail1^+^ or Snail1^−^ cells were counted throughout the heterozygous and homozygous Foxa2 variant embryos. For Fig. 6g, Venus^high^ or Foxa2^high^ cells co-expressing Snail1 and/or Lef1^low/high^ were counted throughout the entire wild-type or Foxa2 variant embryos, excluding the visceral endoderm/definitive endoderm at the surface of the embryo. For Extended Data Fig. 1g, Foxa2, T and Snail1^low/mid/high^-expressing cells in mesoderm, AME and Foxa2^high^ transitory progenitors (based on anatomical position and marker expression) were analysed throughout a complete wild-type embryo, excluding the visceral endoderm/definitive endoderm at the surface of the embryo. For Extended Data Fig. 1i, FVF^+^ epiblast progenitors, FVF, Foxa2^high^ transitory progenitors and Foxa2^high^ visceral endoderm/definitive endoderm (based on anatomical location and marker expression) were quantified for E-cadherin and N-cadherin expression in FVF embryos. In Extended Data Fig. 1h, Foxa2^high^ transitory progenitors, Foxa2^high^ transitory progenitors/visceral endoderm/definitive endoderm and AME (based on anatomical position and marker expression) were analysed for Foxa2, Sox17 and Snail1 expression in wild-type embryos. In Extended Data Fig. 7c, the intensity of E-cadherin was measured in a rectangular area at three different positions within the anterior or posterior epiblast from one *z* plane of FVF embryos. The intensity of Claudin7 was measured in a rectangular area at three different positions within the anterior or posterior epiblast of wild-type embryos.

### Cell culture and differentiation

Mouse ESCs were cultured on mitomycin C-treated mouse embryonic fibroblasts (feeders) in Dulbecco’s modified Eagle’s medium (Life Technologies; 11965092) supplemented with 15% FCS (PAN-Biotech; P30-2602), 0.1 mM β-mercaptoethanol (Life Technologies; 31350-10), 2 mM l-glutamine (Life Technologies; 25030081), 1× non-essential amino acids (Sigma–Aldrich; M7145), 2 mM HEPES (Life Technologies; 15630-056) and 1,000 U ml^−1^ leukaemia inhibitory factor (Sigma–Aldrich; ESG1107). Every 2–3 d, cells were passaged by treatment with 0.05% trypsin (Life Technologies; 25300054) on new feeders and the medium was changed every day. For differentiations of the Snail1 knockout mESCs, T^GFP/+^; Foxa2^tagRFP/+^ mESCs and IDG3.2 mESCs, 1 × 10^5^ cells per 1 cm^2^ were seeded in chemically defined medium, as published recently^65^. After 24 h, endoderm was induced by the addition of 2.5 µM CHIR99021 (Miltenyi Biotec; 130-103-926) for 1 d and 25 ng ml^−1^ activin A (Peprotech; 120-14-300) for all 3 d. For the inhibitor experiments, endoderm was induced as described before, and on days 2 and 3 either 400 ng ml^−1^ DKK1 (Peprotech; 120-30-50) or 1.25 µM IWP2 (Tocris; 3533-10) was supplemented. Differentiations of the FVF mESCs and Foxa2^Venus/Venus^ knockout mESCs were performed as described before^34^. The differentiation of T^GFP/+^; Foxa2^tagRFP/+^ mESCs followed by microarray analysis was carried out based on a previously published protocol^34^. Briefly, before differentiation, Wnt3a feeders were seeded at a density of ~5 × 10^4^ cells per 24 wells. ESCs were passaged onto gelatine-coated plates for 30 min twice to remove remaining feeders from the maintenance cultures. Next, ESCs were transferred to a Wnt3a-expressing feeder plate with a seeding density of 2 × 10^5^ cells per 24 wells. Endoderm differentiation medium, consisting of 500 ml advanced Dulbecco’s modified Eagle’s medium/F-12 (Life Technologies; 12634-10), 500 ml advanced RPMI 1640 (Life Technologies; 12633-012), 22 ml GlutaMAX (Life Technologies; 12860-01), 200 µl AlbuMAX 100 mg ml^−1^ (Life Technologies; 11021-029), 22 ml HEPES 1 M, 70 µl cytidine 150 mg ml^−1^ (Sigma–Aldrich; C4654), 0.9 ml β-mercaptoethanol 50 mM, 12 ml penicillin/streptomycin 10,000 U ml^−1^ (Life Technologies; 10378016) and 1 ml Insulin-Transferrin-Selenium-Ethanolamine (Life Technologies; 51500-056) supplemented with 12 ng ml^−1^ activin A, was used to induce endoderm differentiation. For mesoderm differentiation, 3 ng ml^−1^ activin A and 7.5 ng ml^−1^ Bmp4 were added to the endoderm differentiation medium. The medium was changed every day.

### Affymetrix microarray analysis

RNA was extracted using an miRNeasy Mini Kit (Qiagen; 217004) and total RNA (150 ng) amplified using the Ambion WT Expression Kit and the WT Terminal Labeling Kit (Affymetrix). Amplified complementary DNA was hybridized on Affymetrix Mouse Gene ST 1.0 arrays. Staining and scanning (Fluidics Script FS450_0007) were done according to the Affymetrix expression protocol. An expression console (version 1.2; Affymetrix) was used for quality control. CEL files were imported into R (3.6.3) and robust multichip average normalized using the oligo (1.48.0) package. Data were then pre-filtered and 32,000 probesets with the highest mean expression across all samples were kept for further analysis. Genewise testing for differential expression was performed using limma (3.40.6) and genes with an absolute log_2_[fold change] of >1 and an adjusted *P* < 0.05 were defined as differentially expressed. All microarray data are available from the (NCBI) Gene Expression Omnibus (GEO) under accession GSE148226.

### RNA-seq data analysis

RNA-seq data from Foxa2 knockout definitive endoderm (processed *rsem.genes.results.txt.gz files) were downloaded from the National Center for Biotechnology Information (NCBI) GEO (GSE116262) and analysed in R (3.6.3). RSEM output files were imported into R using the tximport (1.12.3) package and batch correction was performed using the RUVr function (*k* = 1) from the RUVseq (1.18.0) package. Counts were then prepared for DESeq2 (1.20.0) and genes with a minimum mean count below 5 were filtered out. DESeq2 was run with default parameters and log[fold change] shrinkage was performed using apeglm (1.6.0) with the options svalue = TRUE, lfcThreshold = log_2_[1.5]. Differentially expressed genes were defined by an *s* value^66^ of <0.05.

### ChIP-seq data analysis

Foxa2 ChIP-seq raw data (FASTQ files) were downloaded from the NCBI GEO (GSE116262). Trimmomatic (0.39) was used to trim low-quality bases and adapter contaminations, and Bowtie 2 (2.3.5.1) with the --very-sensitive option was used to map reads to the mm10 genome. Peaks were called using GEM (3.4), with the options --k_min 10 --k_max 13, and filtered using *q* value cut-offs of 10^−4^. Binding sites were then mapped to putative target genes by assigning peaks within 20 kilobases of a transcription start site or within a gene body to the respective gene. Genes with Foxa2 binding sites that were regulated in the Foxa2 knockout RNA-seq data were considered to be regulated by Foxa2. The list of genes is provided in Supplementary Table 1.

### Single-cell RNA-seq

Single-cell libraries were generated using the Chromium Single Cell 3′ library and Gel Bead Kit version 2 (10X Genomics; 120237) according to the manufacturer’s instructions. Libraries were sequenced on the HiSeq 4000 (Illumina) with 150-base pair paired-end sequencing of read 2.

### Computational analysis of scRNA-seq data

#### Pre-processing of droplet-based scRNA-seq data

Demultiplexing and alignment to the mm10 mouse genome, identification of unique molecular identifiers (UMIs) and barcode filtering were performed using the Cell Ranger toolkit (version 2.0.0) provided by 10X Genomics. In addition, the velocyto pipeline^67^, which generated a loompy file, was used to determine unspliced and spliced genes. We considered only cells with at least 1,000 expressed genes, where a gene was counted as expressed if we found at least one UMI mapped to it. We further filtered cells with a total UMI count of >125,000 or a fraction of counts from mitochondrial genes of >8%, indicative of stressed or dying cells. We did not apply a minimum library size filter because almost all cells had a total UMI count above 5,000. Cells from all samples were divided by library size, with a target sum of 50,000 UMIs (counts per million normalization; pp.normalize_per_cell in scanpy version 1.4.5.2.dev6+gfa408dc7 in Python 3.7)^68^, and subsequently log + 1 scaled. Then, 3,000 highly variable genes were computed as follows: genes were binned in 20 groups by mean expression and a normalized dispersion was obtained by scaling with the mean and s.d. of the dispersions for genes falling into a given bin for mean expression of genes (pp.highly_variable_genes in scanpy version 1.4.5.2.dev6+gfa408dc7 in Python 3.7 with the flavour cell_ranger to compute normalized dispersions), thereby accounting for differences across batches. Batch-effect correction was done with Scanorama version 1.4 (ref. ^69^) using the corrected feature space. Spliced and unspliced UMI matrices were filtered with a minimum of 20 UMIs each. Then, the data were counts per million normalized and log + 1 scaled. Furthermore, the dataset was restricted to the same highly variable genes as the count matrix.

#### Dimension reduction

We performed our analyses with scanpy version 1.4.5.2.dev6+gfa408dc7 in Python 3.7. First, we computed a principal component analysis (PCA) space with *n* = 50 components and a *k*-nearest neighbour graph on the PCA space with *k* = 30 (tl.pca and pp.neighbors). PCA and UMAP embeddings was computed on the batch-corrected and normalized data with 3,000 highly variable genes. We then used UMAP^70^ to represent the data in the two-dimensional embedding (tl.umap).

#### Clustering and cell-type annotation

The data matrix was clustered with the Louvain algorithm (tl.louvain with resolution 2.0)^71^ and found 27 clusters. All clusters were then inspected for potential substructure and five clusters were further resolved with a low resolution of 0.2. Here, we annotated and merged clusters again according to marker gene expression, resulting in 11 cell stages. Marker gene expression was visualized in a dotplot using scanpy version 1.6.0. Furthermore, we classified the cells according to the cell sorting into FVF^neg^, FVF^low^ and FVF^high^ cells, respectively, determined the amount of Foxa2 mRNA in each cell and classified Foxa2 mRNA-negative and -positive cells based on non-batch-corrected values, where every cell counted as positive with at least one UMI of the Foxa2 gene detected. In FVF^neg^ samples, the number of Foxa2 mRNA-positive cells was 45 out of 4,173 cells and we considered both categories as FVF^neg^.

#### Differential expression analysis

To study differential expression, we used the default scanpy test function (tl.rank_genes_groups with default method t-test_overestim_var and Benjamini–Hochberg correction for multiple testing) to determine the pairwise differences of several populations (that is, epiblast and posterior epiblast, Foxa2 mRNA^+^ and Foxa2 mRNA^−^ posterior epiblast, posterior epiblast and transitory progenitors). We then visualized the top 100 up- and downregulated differentially expressed genes, respectively.

#### Identifying cell differentiation trajectories

To derive cell trajectories, we computed a pseudotemporal ordering using diffusion pseudotime (tl.dpt in scanpy) as a basis for subsequent visualization of gene trajectories towards the endoderm and mesoderm lineage. Next, we used the dynamical model of the scvelo package version 0.2.1 (ref. ^31^) in scanpy version 1.5.1 to determine gene dynamics and potential cell fate decisions towards the endoderm and mesoderm lineage (tl.recover_dynamics and tl.velocity from the scVelo package). To further characterize the cell fate decision process and to leverage the sorting information of the FVF reporter, we used the CellRank package version 1.0.0-rc.0 (ref. ^32^). Specifically, we recomputed the dynamical RNA velocity model excluding extraembryonic tissues and used it in CellRank, which creates a vector field from the data and uses a random walk model to determine initial, intermediate and terminal states in the data (metastable states). We identified six metastable states in the epiblasts (initial), posterior epiblast (intermediate), AME, definitive endoderm, lateral plate mesoderm and nascent endothelium (all terminal). Using the random walk model, we determined a fate probability (that is, the probability of each cell ending up in one of the metastable states). The fate probability of each cell was displayed in a uniform manifold approximation and projection (UMAP) plot, where the colour of the cell was determined by the cluster with the highest fate probability.

To determine the topology of the data and cluster-wise relationships of the cells, we used partition-based graph abstraction (PAGA)^72^ to quantify the connections between the clusters (tl.paga in scanpy version 1.5.1). We display all connections with a scaled connectivity of at least 0.05 (threshold parameter in pl.paga in scanpy) and visualized the fate probability of each cell population as a pie chart (pl.cluster_fates with the mode paga-pie in CellRank). We further determined the differences in fate probability in the posterior epiblast FVF-sorted Foxa2 mRNA-classified cells and displayed the fate probability per group as a bar chart (pl.cluster_fates with the mode bar in CellRank).

We further used CellRank’s compute_lineage_drivers function to determine lineage drivers for each metastable state as the correlation of the gene expression with the respective lineage. Then, we highlighted the correlation with lateral plate mesoderm and definitive endoderm in a scatter plot.

### Statistics and reproducibility

In general, all of the experiments were performed, if possible, with at least three independent biological samples. Sample sizes are provided in the figure captions. Fewer than three independent experiments were used for the FVF embryo sorting and western blot analysis due to the high quantity of embryos required for this experiment. However, a total number of 158 embryos in two independent experiments was used and considered as sufficient. No statistical method was used to predetermine sample size. Data were excluded when immunohistochemical stainings were insufficient, mESC differentiations failed (<2% Foxa2^+^ cells), embryos were ruptured or embryos were at the wrong stage. For the scRNA-seq analysis, one of the FACS sorted FVF^neg^ samples (FVF_neg_3) was excluded from the analysis due to low sequencing depth. The experiments were randomized if possible. The investigators were not blinded to allocation during the experiments and outcome assessment. All replications were successful. The data were analysed using GraphPad Prism software 8 (GraphPad Software).

### Reporting summary

Further information on experimental design is available in the Life Sciences Reporting Summary linked to this article.

## Online content

Any methods, additional references, Nature Research reporting summaries, source data, extended data, supplementary information, acknowledgements, peer review information; details of author contributions and competing interests; and statements of data and code availability are available at 10.1038/s41556-021-00694-x.

## Supplementary information

Reporting Summary

Supplementary Tables Supplementary Table 1: Foxa2 targets. Supplementary Table 2: List of primers used for cloning and genotyping. Supplementary Table 3: List of antibodies used for immunohistochemistry and FACS.

Supplementary Video 1 Supplementary Video 1: Live-cell imaging of a late-streak-stage FVF/SCF embryo. FVF^low^ epiblast progenitors upregulate the fluorescence intensity and leave the epiblast. FVF^high^ transitory progenitors upregulate Sox17 expression and intercalate into the visceral endoderm/definitive endoderm layer.

## Data Availability

The sequencing data that support the findings of this study have been deposited in the Gene Expression Omnibus (GEO) under the accession codes GSE148226 and GSE162534. Previously published sequencing data that were re-analysed here are available under accession codes GSE116262 (samples GSM3223321, GSM3223325, GSM3223326, GSM3223342–GSM3223345, SM3597790 and GSM3597791) Source data are provided with this paper. All other data supporting the findings of this study are available from the corresponding author upon reasonable request.

## Source data

Source Data Fig. 1 Unprocessed western blots. Source Data Fig. 1 Statistical source data. Source Data Fig. 2 Statistical source data. Source Data Fig. 3 Statistical source data. Source Data Fig. 4 Statistical source data. Source Data Fig. 6 Statistical source data. Source Data Fig. 6 Unprocessed western blots. Source Data Extended Data Fig. 1 Statistical source data. Source Data Extended Data Fig. 4 Statistical source data. Source Data Extended Data Fig. 4 Unprocessed western blots. Source Data Extended Data Fig. 5 Unprocessed gels. Source Data Extended Data Fig. 6 Statistical source data. Source Data Extended Data Fig. 7 Statistical source data.

## Code availability

The analyses of scRNA-seq data are available at https://github.com/theislab/gastrulation_analysis.
