# Supplementary material for: Epithelial cell plasticity drives endoderm formation during gastrulation
Source: Nat Cell Biol. 2021 Jun 24;23(7):692–703. doi: 10.1038/s41556-021-00694-x (PMC8277579; doi:10.1038/s41556-021-00694-x)
Supplement: Supplementary file 1 — Reporting Summary [file 41556_2021_694_MOESM1_ESM.pdf]

nature research

Corresponding author(s): Heiko Lickert

Last updated by author(s): Apr 19, 2021

Reporting Summary

Nature Research wishes to improve the reproducibility of the work that we publish. This form provides structure for consistency and transparency in reporting. For further information on Nature Research policies, see Authors & Referees and the Editorial Policy Checklist.

Statistics

For all statistical analyses, confirm that the following items are present in the figure legend, table legend, main text, or Methods section.

n/a	Confirmed	
☐	☒	The exact sample size (*n*) for each experimental group/condition, given as a discrete number and unit of measurement
☐	☒	A statement on whether measurements were taken from distinct samples or whether the same sample was measured repeatedly
☐	☒	The statistical test(s) used AND whether they are one- or two-sided<br>*Only common tests should be described solely by name; describe more complex techniques in the Methods section.*
☒	☐	A description of all covariates tested
☒	☐	A description of any assumptions or corrections, such as tests of normality and adjustment for multiple comparisons
☐	☒	A full description of the statistical parameters including central tendency (e.g. means) or other basic estimates (e.g. regression coefficient) AND variation (e.g. standard deviation) or associated estimates of uncertainty (e.g. confidence intervals)
☐	☒	For null hypothesis testing, the test statistic (e.g. *F*, *t*, *r*) with confidence intervals, effect sizes, degrees of freedom and *P* value noted<br>*Give P values as exact values whenever suitable.*
☒	☐	For Bayesian analysis, information on the choice of priors and Markov chain Monte Carlo settings
☒	☐	For hierarchical and complex designs, identification of the appropriate level for tests and full reporting of outcomes
☒	☐	Estimates of effect sizes (e.g. Cohen's *d*, Pearson's *r*), indicating how they were calculated

Our web collection on statistics for biologists contains articles on many of the points above.

Software and code

Policy information about availability of computer code

Data collection	Gene expression was assessed in differentiated and undifferentiated cells by Affymetrix Mouse Gene ST 1.0 arrays (Applied Biosystems). For flow cytometry BD FACS Aria III was used. Images were acquired with Leica SP5 confocal microscope and Zeiss LSM 880 Airy Scan confocal microscope. Western blots were developed with ChemStudio SA2 Imager from Analytik Jena AG.
Data analysis	Microarray data were analysed using Expression Suite Software v.1.2 Affymetrix (Applied Biosystems) and R (3.6.3)/Bioconductor (3.9) using packages oligo (1.480) and limma (3.40.6) for differential expression analysis. FACS analysis was done via FlowJo™ v10.2. Images were acquired with Leica SP5 confocal microscope and Zeiss LSM 880 Airy Scan confocal microscope. Images taken by Leica confocal were analysed using Leica LAS AF Lite 4.0. Images taken by Zeiss confocal microscope were analysed using Zeiss Zen 2.3 lite Blue software. For quantifications of confocal images and Western Blot ImageJ v1.53c software was used. All statistics were performed using GraphPad Prism software 8 (GraphPad Software Inc., La Jolla, CA). ChIP-seq data were analysed using Trimmomatic (0.39), Bowtie2 (2.3.5.1) and GEM (3.4). ChIP-seq and ATAC-seq data were visulised using bigWigMerge (2) and Gviz (1.28.3). RNA-seq data were analysed using R (3.6.3)/Bioconductor (3.9) and the packages tximport (1.12.3), RUVseq (1.18.0), DESeq2 (1.20.0) and apeglm (1.6.0). Single-cell RNAseq data were analyzed using scanpy versions 1.4.5.2.dev6+gfa408dc7, 1.5.1 and 1.6, anndata version 0.7.1, scanorama version 1.4, gProfiler-official version 1.0.0, scVelo version 0.2.1, CellRank version 1.0.0-rc.0 and Python 3.7 and 3.8. The data analyses of scRNA-seq data are available at https://github.com/theislab/gastrulation_analysis.

For manuscripts utilizing custom algorithms or software that are central to the research but not yet described in published literature, software must be made available to editors/reviewers. We strongly encourage code deposition in a community repository (e.g. GitHub). See the Nature Research guidelines for submitting code & software for further information.

Data

Policy information about availability of data

All manuscripts must include a data availability statement. This statement should provide the following information, where applicable:

- Accession codes, unique identifiers, or web links for publicly available datasets
- A list of figures that have associated raw data
- A description of any restrictions on data availability

All the data generated or analysed during the current study are included in this published article and its supplementary files. The datasets generated during and/or analysed during the current study are available from the corresponding author on reasonable request. All microarray data is available at GEO under the accession number GSE148226 (currently private and only accessible using accession token: uzmbyasspxmttwt). All single cell RNA sequencing data is available under GSE162534 (currently private and only accessible using accession token: ehivmicwztmjncl). In addition, the following public datasets were analyzed that are available in the GEO repository under the accessions GSE116257 (sample GSM3223321), GSE116258 (samples GSM3223325, GSM3223326) and GSE116260 (samples GSM3223342 to GSM3223345, GSM3597790, GSM3597791)"

Field-specific reporting

Please select the one below that is the best fit for your research. If you are not sure, read the appropriate sections before making your selection.

☒ Life sciences ☐ Behavioural & social sciences ☐ Ecological, evolutionary & environmental sciences

For a reference copy of the document with all sections, see nature.com/documents/nr-reporting-summary-flat.pdf

Life sciences study design

All studies must disclose on these points even when the disclosure is negative.

Sample size	No statistical test or power analysis were performed to predetermine sample size. We defined sample sizes based on past experience and based on relevant literature (Mahaddalkar PU, Scheibner K et al., Generation of pancreatic beta cells from CD177+ anterior definitive endoderm, 2020; Böttcher, A., Büttner, M., Tritschler, S. et al. Non-canonical Wnt/PCP signalling regulates intestinal stem cell lineage priming towards enteroendocrine and Paneth cell fates. Nat Cell Biol 23, 23–31 (2021)). In general, all experiments were done, if possible, with at least 3 independent biological samples and sample sizes are provided in figure legends. Less than 3 independent experiments were used for the FVF embryo sorting and WB analysis due to the high quantity of embryos required for this experiment. However, a total number of 158 embryos in two independent experiments was used and considered as sufficient.
Data exclusions	Data was excluded when immunohistochemical stainings were insufficient, mESC differentiations failed (< 2% Foxa2+ cells), embryos were ruptured or at a wrong stage. For scRNA seq analysis, FVF_neg_3 was excluded from analysis due to low sequencing depth.
Replication	Every experiment was repeated a minimum of 3 times, if possible. All replications were successful.
Randomization	There was randomisation done in collection of samples if possible . For microscopy, embryos that were damaged or embedded in a wrong orientation were excluded from anaylysis. However the whole embryos (excluding extra-embryonic) were imaged and not restricted to a specific region. For quantifications embryos were chosen randomly. For mESC differentiation random positions were chosen to take pictures. For mESC differentiations one dish was used per differentiation and all cells differentiated were anaylsed. For FACS analysis a minimum of 10,000 events were randomly analysed. For sc RNA sequencing of FVF embryos a total of 103 early- to late streak stage embryos were randomly chosen and sorted by FACS.
Blinding	The same investigators performed and analysed the experiments, thus no blinding was performed in our studies.

Behavioural & social sciences study design

All studies must disclose on these points even when the disclosure is negative.

Study description	*Briefly describe the study type including whether data are quantitative, qualitative, or mixed-methods (e.g. qualitative cross-sectional, quantitative experimental, mixed-methods case study).*
Research sample	*State the research sample (e.g. Harvard university undergraduates, villagers in rural India) and provide relevant demographic information (e.g. age, sex) and indicate whether the sample is representative. Provide a rationale for the study sample chosen. For studies involving existing datasets, please describe the dataset and source.*
Sampling strategy	*Describe the sampling procedure (e.g. random, snowball, stratified, convenience). Describe the statistical methods that were used to predetermine sample size OR if no sample-size calculation was performed, describe how sample sizes were chosen and provide a rationale for why these sample sizes are sufficient. For qualitative data, please indicate whether data saturation was considered, and what criteria were used to decide that no further sampling was needed.*
Data collection	*Provide details about the data collection procedure, including the instruments or devices used to record the data (e.g. pen and paper,*

Data collection	*computer, eye tracker, video or audio equipment) whether anyone was present besides the participant(s) and the researcher, and whether the researcher was blind to experimental condition and/or the study hypothesis during data collection.*
Timing	*Indicate the start and stop dates of data collection. If there is a gap between collection periods, state the dates for each sample cohort.*
Data exclusions	*If no data were excluded from the analyses, state so OR if data were excluded, provide the exact number of exclusions and the rationale behind them, indicating whether exclusion criteria were pre-established.*
Non-participation	*State how many participants dropped out/declined participation and the reason(s) given OR provide response rate OR state that no participants dropped out/declined participation.*
Randomization	*If participants were not allocated into experimental groups, state so OR describe how participants were allocated to groups, and if allocation was not random, describe how covariates were controlled.*

Ecological, evolutionary & environmental sciences study design

All studies must disclose on these points even when the disclosure is negative.

Study description	*Briefly describe the study. For quantitative data include treatment factors and interactions, design structure (e.g. factorial, nested, hierarchical), nature and number of experimental units and replicates.*
Research sample	*Describe the research sample (e.g. a group of tagged Passer domesticus, all Stenocereus thurberi within Organ Pipe Cactus National Monument), and provide a rationale for the sample choice. When relevant, describe the organism taxa, source, sex, age range and any manipulations. State what population the sample is meant to represent when applicable. For studies involving existing datasets, describe the data and its source.*
Sampling strategy	*Note the sampling procedure. Describe the statistical methods that were used to predetermine sample size OR if no sample-size calculation was performed, describe how sample sizes were chosen and provide a rationale for why these sample sizes are sufficient.*
Data collection	*Describe the data collection procedure, including who recorded the data and how.*
Timing and spatial scale	*Indicate the start and stop dates of data collection, noting the frequency and periodicity of sampling and providing a rationale for these choices. If there is a gap between collection periods, state the dates for each sample cohort. Specify the spatial scale from which the data are taken*
Data exclusions	*If no data were excluded from the analyses, state so OR if data were excluded, describe the exclusions and the rationale behind them, indicating whether exclusion criteria were pre-established.*
Reproducibility	*Describe the measures taken to verify the reproducibility of experimental findings. For each experiment, note whether any attempts to repeat the experiment failed OR state that all attempts to repeat the experiment were successful.*
Randomization	*Describe how samples/organisms/participants were allocated into groups. If allocation was not random, describe how covariates were controlled. If this is not relevant to your study, explain why.*
Blinding	*Describe the extent of blinding used during data acquisition and analysis. If blinding was not possible, describe why OR explain why blinding was not relevant to your study.*

Did the study involve field work? ☐ Yes ☐ No

Field work, collection and transport

Field conditions	*Describe the study conditions for field work, providing relevant parameters (e.g. temperature, rainfall).*
Location	*State the location of the sampling or experiment, providing relevant parameters (e.g. latitude and longitude, elevation, water depth).*
Access and import/export	*Describe the efforts you have made to access habitats and to collect and import/export your samples in a responsible manner and in compliance with local, national and international laws, noting any permits that were obtained (give the name of the issuing authority, the date of issue, and any identifying information).*
Disturbance	*Describe any disturbance caused by the study and how it was minimized.*

Reporting for specific materials, systems and methods

We require information from authors about some types of materials, experimental systems and methods used in many studies. Here, indicate whether each material, system or method listed is relevant to your study. If you are not sure if a list item applies to your research, read the appropriate section before selecting a response.

Materials & experimental systems

n/a	Involved in the study
☐	☒ Antibodies
☐	☒ Eukaryotic cell lines
☐	☐ Palaeontology
☐	☒ Animals and other organisms
☐	☐ Human research participants
☐	☐ Clinical data

Methods

n/a	Involved in the study
☐	☐ ChIP-seq
☐	☒ Flow cytometry
☐	☐ MRI-based neuroimaging

Antibodies

Antibodies used

Mouse CD24-Pacific Blue Biolegend BLD-101819, 1:100
Isotype Control-Pacific Blue, Rat IgG2b, Biolegend, BLD-400627, 1:100
7_AAD Viability Staining Solution, eBioscience, 00-6993, 5μl/ 10^6 cells
Goat SOX17, Acris/Novus, GT15094 1:1000
Rabbit Foxa2, Cell signalling, 8186, 1:1000
Goat Foxa2, Santa Cruz, sc-6554, 1:1000
Mouse E-cadherin, BD, 610181, 1:1000
Rabbit E-cadherin, Cell Signaling, 3195, 1:1000
Mouse N-cadherin, BD, 610920, 1:1000
Chicken GFP, Aves Labs, GFP-1020, 1:1000
Rabbit RFP, Rockland, 600-401-379, 1:1000
Goat Brachyury, Santa Cruz, sc17743, 1:1000
Rabbit Snail1, Cell signaling, C15D3, 1:500
Rabbit Lef1, Abcam, ab137872, 1:500
Rabbit Hsp90, Cell signaling, 4874S, 1:1000
Rat E-Cadherin, TaKaRa, M108, 1:500
Rat Cer1, R&D, MAB1986, 1:500
Rabbit Claudin7, LIFE Technologies, 34-9100, 1:500
Mouse Foxa2, GeneTex, GTX84485, 1:500
Rat Laminin, Millipore, MAB1914, 1:500-1:1000
Rat Laminin, Abcam, ab44941, 1:500-1:1000
Rabbit Collagen 4, Abcam, ab6586, 1:1000
Rabbit EBP50, ab3452, 1:300
Rabbit Ezrin/Radixin/Moesin, Cell signaling, 3141, 1:300
Rabbit Scribble, Santa Cruz, sc-28737, 1:300
donkey anti-goat IgG 555, Invitrogen, A21432, 1:800
donkey anti-rat IgG 647, Dianova, 712-605-150, 1:800
Goat Anti-Rabbit IgG (H+L), HRP Dianova/Jackson, 111-035-144, 1:5000
donkey anti-mouse IgG 488, Invitrogen, A21202, 1:800
donkey anti-rabbit IgG 555, Invitrogen, A31572, 1:800
donkey anti-goat IgG 488, Invitrogen, A11055, 1:800
donkey anti-rabbit IgG 488, Invitrogen, A21206, 1:800
donkey anti-chicken IgY, Dianova, 703-225-155,1:800
donkey anti-mouse IgG 555, Invitrogen, A31570, 1:800

Validation

All primary antibodies were validated by the manufactures. Further, their expression was tested on undifferentiated cells and/or mouse embryos. Various antibody dilutions were tested including the manufacturer's recommended dilution. The secondary antibody only controls/isotype controls were used to evaluate and confirm specificity of the antibodies to the respective epitopes. All of the antibodies used in this study have been used and reported in prior studies.

primary antibodies:
Mouse CD24-Pacific Blue Biolegend BLD-101819 - https://www.biolegend.com/en-us/products/pacific-blue-anti-mouse-cd24-antibody-3584
Isotype Control-Pacific Blue, Rat IgG2b, Biolegend, BLD-400627 - https://www.biolegend.com/en-us/products/pacific-blue-rat-igg2b-kappa-isotype-ctrl-3159?GroupID=GROUP29
7_AAD Viability Staining Solution, eBioscience, 00-6993 - https://www.fishersci.ca/shop/products/7-aad-viability-staining-solution/501128859
Goat SOX17, Acris/Novus, GT15094 - https://www.novusbio.com/products/sox17-antibody_af1924
Rabbit Foxa2, Cell signalling, 8186 - https://www.cellsignal.com/products/primary-antibodies/foxa2-hnf3b-d56d6-xp-rabbit-mab/8186
Goat Foxa2, Santa Cruz, sc-6554 - https://www.scbt.com/p/hnf-3beta-antibody-m-20
Mouse E-cadherin, BD, 610181 - https://www.bdbiosciences.com/us/applications/research/stem-cell-research/cancer-research/human/purified-mouse-anti-e-cadherin-36e-cadherin/p/610181

Rabbit E-cadherin, Cell Signaling, 3195 - https://www.cellsignal.com/products/primary-antibodies/e-cadherin-24e10-rabbit-mab/3195
Mouse N-cadherin, BD, 610920 - https://www.bdbiosciences.com/us/applications/research/stem-cell-research/cancer-research/human/purified-mouse-anti-n-cadherin-32n-cadherin/p/610920
Chicken GFP, Aves Labs, GFP-1020 - https://www.aveslabs.com/products/anti-green-fluorescent-protein-antibody-gfp
Rabbit RFP, Rockland, 600-401-379 - https://rockland-inc.com/store/Antibodies-to-GFP-and-Antibodies-to-RFP-600-401-379-O4L_24299.aspx
Goat Brachyury, Santa Cruz, sc17743 - https://www.scbt.com/p/brachyury-antibody-n-19
Rabbit Snail1, Cell signaling, C15D3 - https://www.cellsignal.com/products/primary-antibodies/snail-c15d3-rabbit-mab/3879
Rabbit Lef1, Abcam, ab137872 - https://www.abcam.com/lef1-antibody-epr2029y-ab137872.html
Rabbit Hsp90, Cell signaling, 4874S - https://www.cellsignal.com/products/primary-antibodies/hsp90-antibody/4874
Rat E-Cadherin, TaKaRa, M108 - https://www.takarabio.com/products/antibodies-and-elisa/primary-antibodies-and-elisas-by-research-area/cell-adhesion-and-ecm/cadherin
Rat Cer1, R&D, MAB1986 - https://www.rndsystems.com/products/mouse-cerberus-1-antibody-225807_mab1986
Rabbit Claudin7, LIFE Technologies, 34-9100 - https://www.thermofisher.com/antibody/product/Claudin-7-Antibody-Polyclonal/34-9100
Mouse Foxa2, GeneTex, GTX84485 - https://www.genetex.com/Product/Detail/FOXA2-antibody-3C10/GTX84485
Rat Laminin, Millipore, MAB1914 - https://www.merckmillipore.com/DE/de/product/Anti-Laminin-gamma-1-Antibody-clone-A5,MM_NF-MAB1914P?ReferrerURL=https%3A%2F%2Fwww.google.com%2F&bd=1
Rat Laminin, Abcam, ab44941 - https://www.abcam.com/laminin-beta-1-antibody-lt3-ab44941.html
Rabbit Collagen 4, Abcam, ab6586 - https://www.abcam.com/collagen-iv-antibody-ab6586.html
Rabbit EBP50, ab3452 - https://www.abcam.com/ebp50nherf-1-antibody-ab3452.html
Rabbit Ezrin/Radixin/Moesin, Cell signaling, 3141 - https://www.cellsignal.com/products/primary-antibodies/phospho-ezrin-thr567-radixin-thr564-moesin-thr558-antibody/3141
Rabbit Scribble, Santa Cruz, sc-28737 - https://www.scbt.com/p/scrib-antibody-h-300

secondary antibodies:
donkey anti-goat IgG 555, Invitrogen, A21432 - https://www.thermofisher.com/antibody/product/Donkey-anti-Goat-IgG-H-L-Cross-Adsorbed-Secondary-Antibody-Polyclonal/A-21432
donkey anti-rat IgG 647, Dianova, 712-605-150 - https://www.dianova.com/en/shop/712-605-150-donkey-igg-anti-rat-igg-hl-alexa-fluor-647-minx-bockgogphshohurbsh/
Goat Anti-Rabbit IgG (H+L), HRP Dianova/Jackson, 111-035-144 - https://www.dianova.com/shop/111-035-144-ziege-igg-anti-kaninchen-igg-hl-hrpo-minx-humsrt/
donkey anti-mouse IgG 488, Invitrogen, A21202 - https://www.thermofisher.com/order/genome-database/generatePdf?productName=Mouse%20IgG%20(H+L)&assayType=PRANT&productId=A-21202&detailed=true
donkey anti-rabbit IgG 555, Invitrogen, A31572 - https://www.thermofisher.com/antibody/product/Donkey-anti-Rabbit-IgG-H-L-Highly-Cross-Adsorbed-Secondary-Antibody-Polyclonal/A-31572
donkey anti-goat IgG 488, Invitrogen, A11055 - https://www.thermofisher.com/antibody/product/Donkey-anti-Goat-IgG-H-L-Cross-Adsorbed-Secondary-Antibody-Polyclonal/A-11055
donkey anti-rabbit IgG 488, Invitrogen, A21206 - https://www.thermofisher.com/antibody/product/Donkey-anti-Rabbit-IgG-H-L-Highly-Cross-Adsorbed-Secondary-Antibody-Polyclonal/A-21206
donkey anti-chicken IgY, Dianova, 703-225-155 - https://www.dianova.com/downloads/Jackson/703-225-155.pdf
donkey anti-mouse IgG 555, Invitrogen, A31570 - https://www.thermofisher.com/order/genome-database/generatePdf?productName=Mouse%20IgG%20(H+L)&assayType=PRANT&productId=A-31570&detailed=true

Eukaryotic cell lines

Policy information about cell lines

Cell line source(s)	The cell lines used are TGFP mESCs (Fehling et al., 2003), TGFP/+;Foxa2tagRFP/+ mESC were generated in the lab, FVF mESCs (Burtscher et al., 2013), Snail1 KO mESCs were generated in the lab. IDG3.2 mESCs (Hitz et al., 2007), Foxa2 KO mESCs (Cernilogar et al. 2019).
Authentication	No authentication was done.
Mycoplasma contamination	All cell lines were mycoplasma negative
Commonly misidentified lines (See ICLAC register)	No misidentified cell lines were used.

Palaeontology

Specimen provenance	*Provide provenance information for specimens and describe permits that were obtained for the work (including the name of the issuing authority, the date of issue, and any identifying information).*
Specimen deposition	*Indicate where the specimens have been deposited to permit free access by other researchers.*

Dating methods | *If new dates are provided, describe how they were obtained (e.g. collection, storage, sample pretreatment and measurement), where they were obtained (i.e. lab name), the calibration program and the protocol for quality assurance OR state that no new dates are provided.*

☐ Tick this box to confirm that the raw and calibrated dates are available in the paper or in Supplementary Information.

Animals and other organisms

Policy information about studies involving animals; ARRIVE guidelines recommended for reporting animal research

Laboratory animals: Mus musculus: CD-1, mTmG reporter (C57Bl/6J; Muzumdar et al., 2007), FVF (C57Bl/6J; Burtscher et al., 2013), SCF reporter (C57Bl/6J; Burtscher et al., 2012). Age of females: ≥ 6 weeks, age of males: ≥ 8 weeks.

Wild animals: No wild animals were used in this study.

Field-collected samples: No field-collected samples were used in this study.

Ethics oversight: Animal experiments were performed at the central facilities at HMGU in accordance with the German animal welfare legislation and acknowledged guidelines of the Society of Laboratory Animals (GV-SOLAS) and of the Federation of Laboratory Animal Science Associations (FELASA).

Note that full information on the approval of the study protocol must also be provided in the manuscript.

Human research participants

Policy information about studies involving human research participants

Population characteristics: *Describe the covariate-relevant population characteristics of the human research participants (e.g. age, gender, genotypic information, past and current diagnosis and treatment categories). If you filled out the behavioural & social sciences study design questions and have nothing to add here, write "See above."*

Recruitment: *Describe how participants were recruited. Outline any potential self-selection bias or other biases that may be present and how these are likely to impact results.*

Ethics oversight: *Identify the organization(s) that approved the study protocol.*

Note that full information on the approval of the study protocol must also be provided in the manuscript.

Clinical data

Policy information about clinical studies

All manuscripts should comply with the ICMJE guidelines for publication of clinical research and a completed CONSORT checklist must be included with all submissions.

Clinical trial registration: *Provide the trial registration number from ClinicalTrials.gov or an equivalent agency.*

Study protocol: *Note where the full trial protocol can be accessed OR if not available, explain why.*

Data collection: *Describe the settings and locales of data collection, noting the time periods of recruitment and data collection.*

Outcomes: *Describe how you pre-defined primary and secondary outcome measures and how you assessed these measures.*

ChIP-seq

Data deposition

☐ Confirm that both raw and final processed data have been deposited in a public database such as GEO.

☐ Confirm that you have deposited or provided access to graph files (e.g. BED files) for the called peaks.

Data access links
May remain private before publication.: *For "Initial submission" or "Revised version" documents, provide reviewer access links. For your "Final submission" document, provide a link to the deposited data.*

Files in database submission: *Provide a list of all files available in the database submission.*

Genome browser session
(e.g. UCSC): *Provide a link to an anonymized genome browser session for "Initial submission" and "Revised version" documents only, to enable peer review. Write "no longer applicable" for "Final submission" documents.*

Methodology

Replicates: *Describe the experimental replicates, specifying number, type and replicate agreement.*

Sequencing depth	*Describe the sequencing depth for each experiment, providing the total number of reads, uniquely mapped reads, length of reads and whether they were paired- or single-end.*
Antibodies	*Describe the antibodies used for the ChIP-seq experiments; as applicable, provide supplier name, catalog number, clone name, and lot number.*
Peak calling parameters	*Specify the command line program and parameters used for read mapping and peak calling, including the ChIP, control and index files used.*
Data quality	*Describe the methods used to ensure data quality in full detail, including how many peaks are at FDR 5% and above 5-fold enrichment.*
Software	*Describe the software used to collect and analyze the ChIP-seq data. For custom code that has been deposited into a community repository, provide accession details.*

Flow Cytometry

Plots

Confirm that:

☒ The axis labels state the marker and fluorochrome used (e.g. CD4-FITC).

☒ The axis scales are clearly visible. Include numbers along axes only for bottom left plot of group (a 'group' is an analysis of identical markers).

☒ All plots are contour plots with outliers or pseudocolor plots.

☒ A numerical value for number of cells or percentage (with statistics) is provided.

Methodology

Sample preparation	Single cell suspensions of undifferentiated and/or S1-S7 differentiated mouse ES cells were fixed, permeabilized and stained with the mentioned primary and secondary antibodies or conjugated antibodies and their isotype controls.
Instrument	BD FACS Aria III
Software	FlowJo v10.2
Cell population abundance	FACS sorting was performed on differentiated T-GFP/Foxa2-RFP mESC. These cells were sorted directly in QIAzol Lysis Reagent for RNA extraction for Affymetrix microarray analysis.
Gating strategy	In the preliminary FSC/SSC plot the gates were placed surrounding the main population and to gate for positive/negative populations gatings were put based on the Isotype or secondary antibody control.

☒ Tick this box to confirm that a figure exemplifying the gating strategy is provided in the Supplementary Information.

Magnetic resonance imaging

Experimental design

Design type	*Indicate task or resting state; event-related or block design.*
Design specifications	*Specify the number of blocks, trials or experimental units per session and/or subject, and specify the length of each trial or block (if trials are blocked) and interval between trials.*
Behavioral performance measures	*State number and/or type of variables recorded (e.g. correct button press, response time) and what statistics were used to establish that the subjects were performing the task as expected (e.g. mean, range, and/or standard deviation across subjects).*

Acquisition

Imaging type(s)	*Specify: functional, structural, diffusion, perfusion.*
Field strength	*Specify in Tesla*
Sequence & imaging parameters	*Specify the pulse sequence type (gradient echo, spin echo, etc.), imaging type (EPI, spiral, etc.), field of view, matrix size, slice thickness, orientation and TE/TR/flip angle.*
Area of acquisition	*State whether a whole brain scan was used OR define the area of acquisition, describing how the region was determined.*

Diffusion MRI ☐ Used ☐ Not used

Preprocessing

Preprocessing software	*Provide detail on software version and revision number and on specific parameters (model/functions, brain extraction, segmentation, smoothing kernel size, etc.).*
Normalization	*If data were normalized/standardized, describe the approach(es): specify linear or non-linear and define image types used for transformation OR indicate that data were not normalized and explain rationale for lack of normalization.*
Normalization template	*Describe the template used for normalization/transformation, specifying subject space or group standardized space (e.g. original Talairach, MNI305, ICBM152) OR indicate that the data were not normalized.*
Noise and artifact removal	*Describe your procedure(s) for artifact and structured noise removal, specifying motion parameters, tissue signals and physiological signals (heart rate, respiration).*
Volume censoring	*Define your software and/or method and criteria for volume censoring, and state the extent of such censoring.*

Statistical modeling & inference

Model type and settings	*Specify type (mass univariate, multivariate, RSA, predictive, etc.) and describe essential details of the model at the first and second levels (e.g. fixed, random or mixed effects; drift or auto-correlation).*
Effect(s) tested	*Define precise effect in terms of the task or stimulus conditions instead of psychological concepts and indicate whether ANOVA or factorial designs were used.*

Specify type of analysis: ☐ Whole brain ☐ ROI-based ☐ Both

Statistic type for inference (See Eklund et al. 2016)	*Specify voxel-wise or cluster-wise and report all relevant parameters for cluster-wise methods.*
Correction	*Describe the type of correction and how it is obtained for multiple comparisons (e.g. FWE, FDR, permutation or Monte Carlo).*

Models & analysis

n/a	Involved in the study
☐	☐ Functional and/or effective connectivity
☐	☐ Graph analysis
☐	☐ Multivariate modeling or predictive analysis

Functional and/or effective connectivity	*Report the measures of dependence used and the model details (e.g. Pearson correlation, partial correlation, mutual information).*
Graph analysis	*Report the dependent variable and connectivity measure, specifying weighted graph or binarized graph, subject- or group-level, and the global and/or node summaries used (e.g. clustering coefficient, efficiency, etc.).*
Multivariate modeling and predictive analysis	*Specify independent variables, features extraction and dimension reduction, model, training and evaluation metrics.*
