# Supplementary figures and images for: Epithelial cell plasticity drives endoderm formation during gastrulation
Source: Nat Cell Biol. 2021 Jun 24;23(7):692–703. doi: 10.1038/s41556-021-00694-x (PMC8277579; doi:10.1038/s41556-021-00694-x)

**a**  $n = 122$  FVF embryos

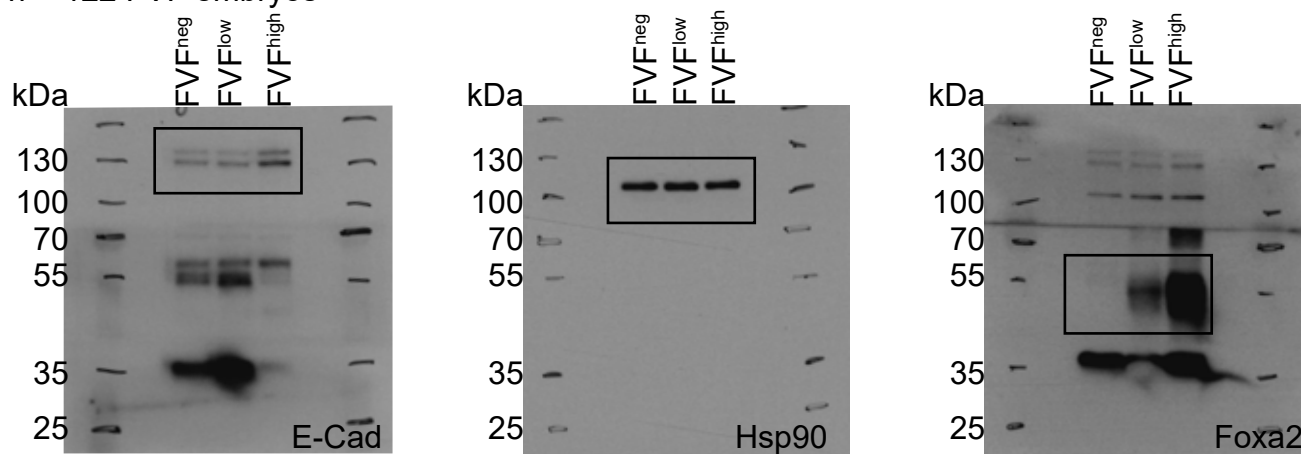

**b**  $n = 36$  FVF embryos

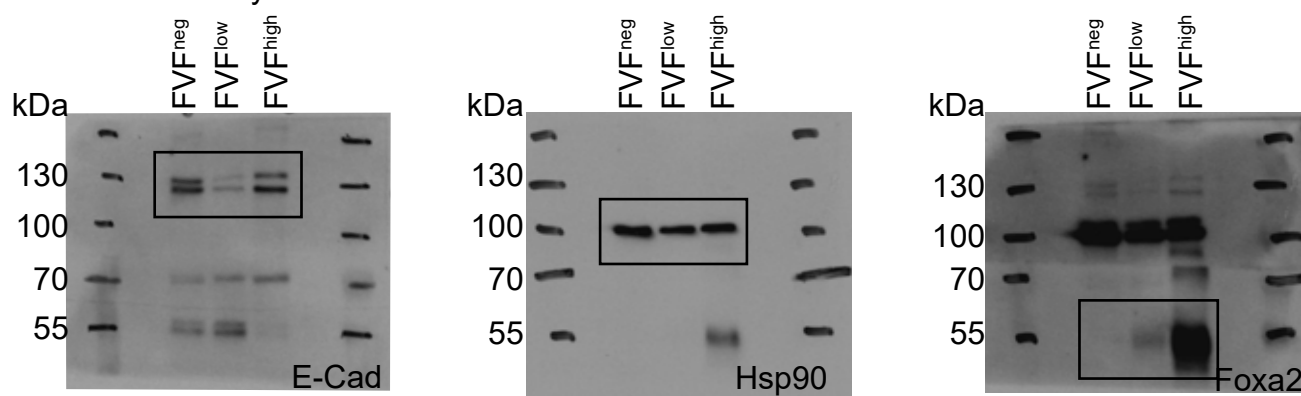

Supplement: Source Data Fig. 1 — Unprocessed western blots. [file 41556_2021_694_MOESM4_ESM.pdf]

**a**  $n = 122$  FVF embryos

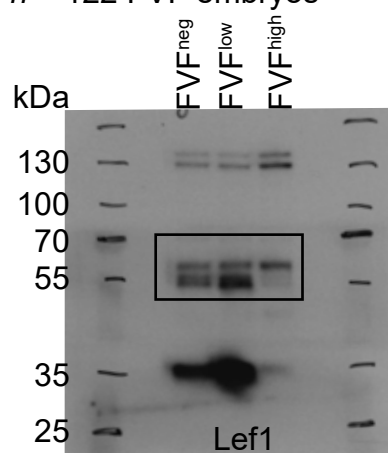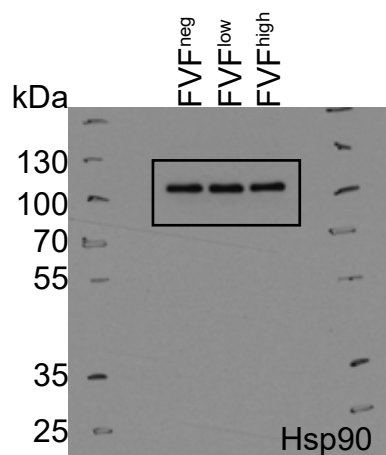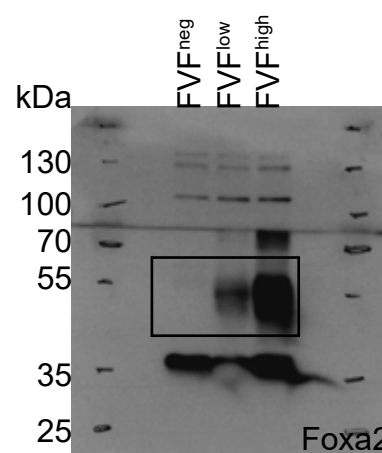

**b**  $n = 36$  FVF embryos

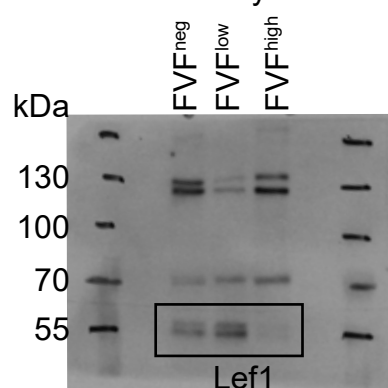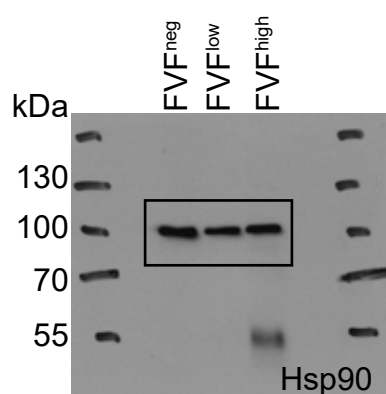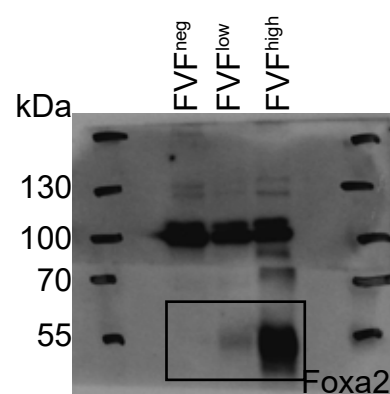

Supplement: Source Data Fig. 6 — Unprocessed western blots. [file 41556_2021_694_MOESM10_ESM.pdf]

**a** Foxa2<sup>Venus/+</sup> mESCs day 3

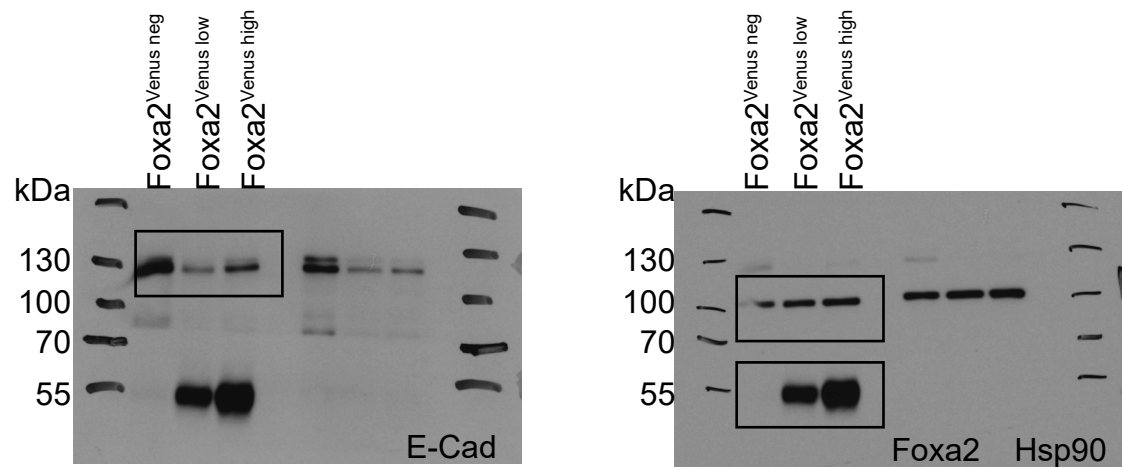

**b** Foxa2<sup>Venus/+</sup> mESCs day 3

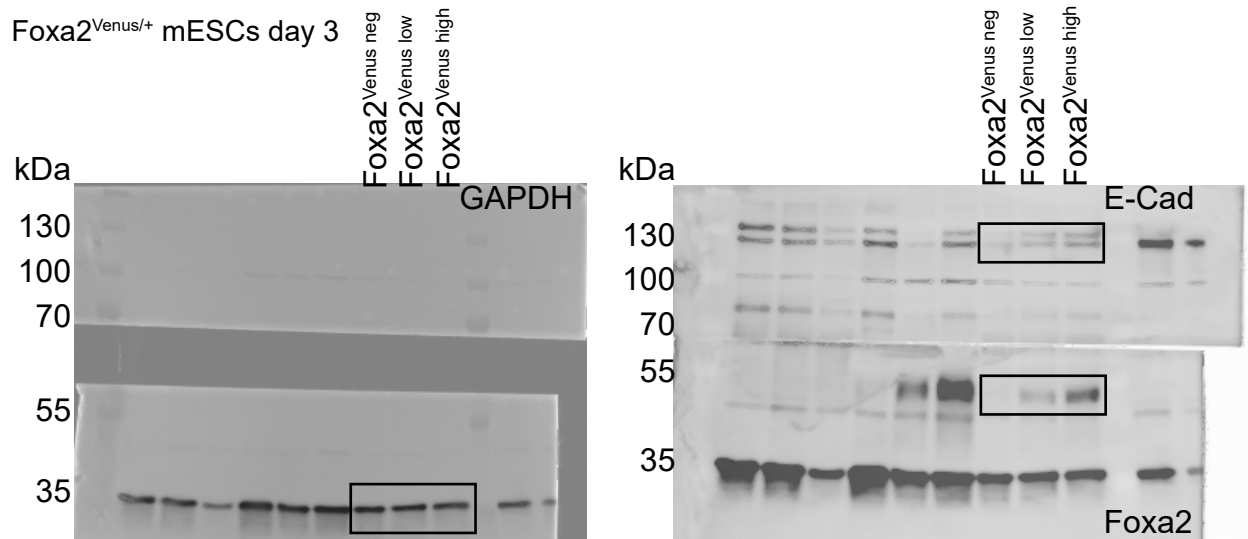

Supplement: Source Data Extended Data Fig. 4 — Unprocessed western blots. [file 41556_2021_694_MOESM13_ESM.pdf]

# Source Data: Extended Data Fig. 5b

**a**

5' genotyping PCR

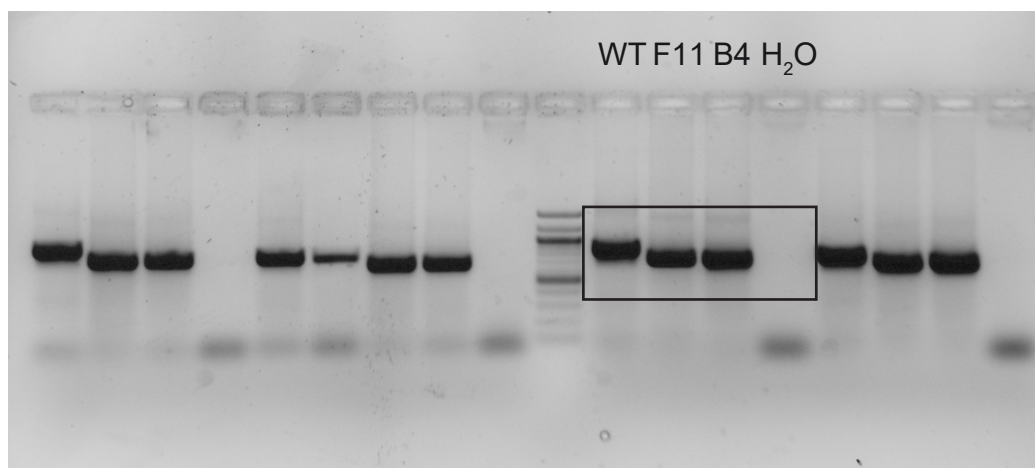

**b**

3' genotyping PCR

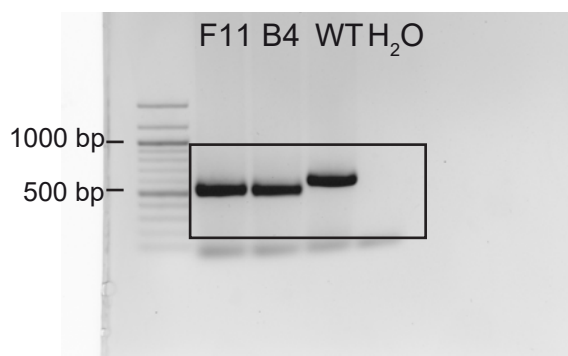

Supplement: Source Data Extended Data Fig. 5 — Unprocessed gels. [file 41556_2021_694_MOESM14_ESM.pdf]
